# Systematic review on community-based interventions targeting prevention of overweight and obesity in children and adolescents

**DOI:** 10.3389/fpubh.2025.1687963

**Published:** 2025-10-29

**Authors:** Júlia Santamaria, Nikolai Mühlberger, Daniela Schmid, Igor Kuchin, Erica A. Suzumura, Nardeen Ayad, Yuli Lily Hsieh, Kieran Tuohy, Michael Laxy, Uwe Siebert, Beate Jahn

**Affiliations:** ^1^Department of Public Health, Health Services Research and Health Technology Assessment, Institute of Public Health, Medical Decision Making and Health Technology Assessment, UMIT TIROL-University for Health Sciences and Technology, Hall in Tirol, Austria; ^2^Faculty of Life Sciences, Albstadt-Sigmaringen University, Sigmaringen, Germany; ^3^Department of Preventive Medicine, Faculdade de Medicina da Universidade de São Paulo FMUSP, São Paulo, Brazil; ^4^Department of Pediatrics, Washington University, Saint Louis, MO, United States; ^5^Interfaculty Initiative in Health Policy, Harvard University, Cambridge, MA, United States; ^6^Department of Health Policy and Management, Center for Health Decision Science, Harvard T.H. Chan School of Public Health, Boston, MA, United States; ^7^Nutrition and Nutrigenomics Unit, Research and Innovation Cente-Fondazione Edmund Mach, San Michele all'Adige, Italy; ^8^School of Food Science and Nutrition, University of Leeds, Leeds, United Kingdom; ^9^Public Health and Prevention, School of Medicine and Health, Technical University of Munich, Munich, Germany; ^10^Department of Epidemiology, Harvard T.H. Chan School of Public Health, Boston, MA, United States; ^11^Institute for Technology Assessment and Department of Radiology, Massachusetts General Hospital, Harvard Medical School, Boston, MA, United States

**Keywords:** community-based intervention, obesity and overweight, prevention, environmental approaches, whole-system approach

## Abstract

**Background:**

With increasing obesity rates, community-based interventions (CBIs) have gained attention as more evidence suggests that the environment has a significant impact on individuals’ behaviors. We aimed to conduct a systematic review of CBIs for preventing overweight/obesity in children and adolescents.

**Subject and methods:**

We searched PubMed (January 2011–December 2024) for studies evaluating CBIs for the prevention of overweight and obesity. We included controlled interventional studies that reported weight-related outcomes. The assessment of risks of bias of the included studies was performed using the Cochrane Collaboration tool. Results are reported in systematic evidence tables, including information on main study characteristics and effect sizes.

**Results:**

A total of 2,724 articles were retrieved, of which 37 publications representing 28 projects from seven world regions were included for review. Most of the interventions targeted children. Reported intervention effects ranged from none to small effects. A beneficial intervention effect was observed in 11 out of 16 studies that calculated the intervention effect for BMI z-score and in seven out of nine studies for the prevalence of overweight/obesity. Effect sizes ranged from −0.26 to −0.03 for the BMI z-score and up to an adjusted odds ratio of 0.65 in the intervention group compared to the control group, with respect to the prevalence of overweight/obesity. The risk of bias of the studies was moderate to high.

**Conclusion:**

Overall, our study found inconclusive evidence on the benefits of CBIs. While creating health-promoting environments can influence population-wide nutrition and physical activity behaviors by making healthy choices more accessible, methodological challenges exist to accurately capture the true effects of CBIs.

## 1 Introduction

The prevalence of obesity worldwide has tripled since the 1970s, rising at alarming rates and becoming a growing public health concern (1–3). In 2022, 2.5 billion adults and over 390 million children and adolescents (5–19 years old) worldwide were overweight or obese (2). Obesity and overweight are related to a wide range of co-morbidities leading to increased mortality and reduced quality of life, resulting in productivity losses and a huge economic burden for healthcare systems (1, 4). To combat the increasing rates of obesity, interventions to prevent obesity in the general population are of high public health importance. Several action plans at international and national levels have been implemented to reduce weight-related non-communicable diseases and to promote healthy diets and physical activity through health promotion programs (5).

Individual behaviors and lifestyles are greatly influenced by the environment and social contexts in which individuals live (5). Swinburn coined the term “obesogenic environments” in the 1990s, defined as the contexts or surroundings that foster unhealthy eating and physical inactivity (6, 7). Based on this rationale and given the complexity of the etiology of obesity, community-based interventions (CBIs) have been recommended to prevent obesity (8–13), shifting the focus from interventions at the individual level to population-based interventions (14).

CBIs are comprehensive upstream strategies that target different aspects of the environment that influence its residents (15). They comprise multiple components in multiple settings and rely on community participation and the collaboration of community stakeholders during the process of planning and implementation (16–19). A CBI was first implemented in the North Karelia Project (Finland) in the 1970s to tackle cardiovascular diseases. CBIs were thereafter adopted to address several other public health issues over the past decades as well (19), including unhealthy diet and physical inactivity. However, due to the extensive nature of CBIs, the effects in preventing and reducing the targeted diseases are small (8, 19, 20). Additionally, assessing the effect of these interventions can be methodologically challenging due to selection bias, small sample sizes, and the lack of appropriate control groups to measure population-level change. Furthermore, randomization can be infeasible in many settings (19, 20). Continuous monitoring of CBIs and process evaluations is required to better understand key aspects for the success of such interventions (17).

Except for a systematic review by Wolfenden et al. (20), previous systematic reviews (9, 12, 21) have limited their searches to childhood obesity prevention. However, in the review conducted by Wolfenden et al. (20), the authors could not identify any study that collected data from adults, underscoring the scarcity of studies evaluating CBIs for obesity prevention in adulthood. Our systematic review aimed to update the search of Wolfenden et al. (20), covering the period from 2011 to the present, to identify and synthesize the characteristics and most recent evidence of CBIs for obesity prevention targeting children, adolescents, and/or adults, with a focus on the effects on weight-related outcomes.

## 2 Methods

We performed a systematic review on CBIs to prevent overweight and obesity following the recommendations of the Cochrane Handbook for Systematic Review of Interventions (22). Reporting of the systematic review was based on the Preferred Reporting Items for Systematic Reviews and Meta-Analyses (PRISMA) guidelines (23).

### 2.1 Study selection

We searched for studies evaluating a CBI for obesity prevention that targeted the general population (adults, adolescents and/or children) from middle- or high-income countries (6, 24). The interventions of interest were defined as any intervention targeting an entire community with the main goal of changing physical activity and nutrition to prevent overweight and obesity. The interventions had to include strategies implemented in more than one setting within the community, involve community participation, and have stakeholders in the planning or implementation of the intervention. Only randomized and quasi-experimental designs (non-randomized controlled trials) with an intervention duration of at least 12 months were included. It was required that the intervened community be compared to a control group that received no intervention or another CBI. Further inclusion criteria were the reporting of at least one quantitative weight-related outcome: BMI (Body Mass Index), percentile BMI (PBMI), BMI standardized score (BMI z-score), or prevalence of overweight and obesity. Only articles published in English, German, or Spanish in peer-reviewed journals were assessed. We excluded studies if (1) they targeted people with overweight or obesity only, or only people with other pre-existing medical conditions; (2) interventions were only implemented in one setting; (3) they did not have a control group; (4) they did not measure quantitative weight-related outcomes, (5) their main aim was treating, rather than preventing, overweight and obesity (see Supplementary Table S1).

### 2.2 Search strategy

Based on these criteria, a search strategy was developed using a mix of keywords and MeSH terms related to “community,” “intervention,” “prevention,” and “obesity,” combined with Boolean operators (Supplementary Table S2). The search was conducted in PubMed’s electronic database and was limited to studies published between January 2011 (endpoint of the last systematic review by Wolfenden et al.) and December 2024. Additionally, we reviewed the articles included in previous reviews to identify studies relevant to our review that may have been missed (20, 25). Studies published before 2011 were retrieved from the previous systematic review by Wolfenden et al. (20) and added to our results.

### 2.3 Screening

After retrieving the publications identified by our search terms from the database, duplicates were identified and removed. The remaining publications underwent a first screening based on title and abstract, followed by a full-text examination, which provided the final selection of studies to be included in the systematic review. The full-text screening was performed by two independent reviewers (JS, ES). Disagreements were resolved between the two reviewers, and a third reviewer (BJ, NM) was consulted for a final decision.

### 2.4 Data extraction and synthesis

A standardized extraction form was developed before the review to collect relevant information from the studies. The results were summarized in two systematic evidence tables (available in the Supplementary Material). The first evidence table (see Supplementary Table S3) provides information about the study characteristics, intervention components, community characteristics, and target populations. The second evidence table (see Supplementary Table S4) provides information related to the outcomes and effectiveness of the intervention. A third table (see Table 1) summarizes the information from both previous tables and was created to present the most relevant data to include in the main manuscript. The primary outcomes of interest for our review were quantitative weight-related outcomes: BMI, BMI z-scores for children (expressed as the number of standard deviations or Z-scores below or above the reference standard mean, adjusted for age and sex), and prevalence of overweight/obesity. These standardized assessments of weight status and BMI are excellent predictors of a higher risk of disease (26–28). Differences between baseline and follow-up measurements were calculated by the reviewers when not reported explicitly. The information reported in the evidence tables was summarized in a narrative synthesis. For some studies, more than one publication was available. If multiple publications reported on the same study, they were summarized at the study level.

**Table 1:** Study characteristics and results of the included studies.

Authors, years, project name, country	Study design, target population, and type of communities	Intervention settings, duration, and data collection points	Number of communities and participants	Effect size: intervention effect (intervention vs. control)
Coffield et al. (2015) (33) Economos et al. (2007) (69) Economos et al. (2013) (70) Shape Up Somerville (SUS); Massachusetts, USA	Quasi-experimental, non-randomized controlled trial Children in grades 1–3 in elementary schools and parents of children attending SUS schools Urban communities	Elementary schools, community spaces and facilities, and homes 3 years (2002–2004/05) Baseline, 1-year, and 2-year follow-up	**Control** Baseline: 2 communities: 20 schools (15 in Control 1, 5 in Control 2): *n* = 890 children analyzed, *n* = 1,003 parents of 567 households analyzed Follow-up: *n* = 793 children analyzed (*n* = 561 in Control 1, *n* = 232 in Control 2) at year 1, and *n* = 693 at year 2, *n* = 507 parents analyzed at year 2 **Intervention** Baseline: 1 community: 10 schools: *n* = 461 children analyzed, *n* = 527 parents of 293 households Follow-up: *n* = 385 children analyzed at year 1, *n* = 335 children at year 2, *n* = 162 parents analyzed at year 2	1) BMI z-score adjusting for the covariates and pooling the two control communities: −0.1005 (95% CI −0.1541, −0.0555; *p* = 0.001) at 1 year, −0.06 (95% CI −0.08, −0.04; *p* = 0.005) at 2 years 2) Prevalence of overweight/obesity: Overall: aOR 0.71 (95% CI 0.56, 0.90; *p* = 0.004); males: 0.61 (*p* = 0.01); females: 0.78 (*p* = 0.01) at 2 years Covariates included sex, age, grade, primary language, race/ethnicity, child BMI z-score at baseline, and clustering on community. Statistically significant intervention effects in the reduction of BMI z-scores and overweight/obesity prevalence after 1 and 2 years.
Bell et al. (2019) (38) Obesity Prevention and Lifestyle (OPAL); South Australia	Quasi-experimental repeated cross-sectional design, non-randomized Children (4–5 years old, 9–11 years old, 14–16 years old) Disadvantaged communities (low SES according to the Index of Relative Socioeconomic Disadvantage-IRSD-)	Community spaces and facilities, workplaces, and schools 2–3 years Baseline and 5-year follow-up The evaluation period was shortened due to initial delays to a 2–3 year intervention	**Control** Baseline: 12 lower SES (Index of Relative Socioeconomic Disadvantage-IRSD) communities (phase 1) and eight communities (phase 2): *n* = 1,238 completed surveys, *n* = 1,145 for measurements Follow-up: 6 communities (phase 1) and four communities (phase 2): *n* = 781 completed surveys, *n* = 750 for measurements **Intervention** Baseline: 6 communities (phase 1) and four communities (phase 2): *n* = 1,373 completed surveys, *n* = 1,208 for measurements Follow-up: 6 communities (phase 1) and four communities (phase 2): *n* = 1,092 completed surveys, *n* = 1,010 for measurements	1) BMI z-score: −0.08 (95% CI −0.24, 0.08; *p* = 0.31) (after Bonferroni adjustment, marginal mean difference adjusted for SES) 2) Prevalence of overweight: aOR 0.87 (95% CI 0.6, 1.25; *p* > 0.05) (after Bonferroni adjustment, adjusted for age and SES) 3) Prevalence of obesity: aOR 0.51 (95% CI 0.28, 0.92; *p* > 0.05) (after Bonferroni adjustment, adjusted for age and SES) No statistically significant differences in the likelihood of obesity and BMI z-score, but the intervention group was favored in these outcomes.
Gomez et al. (2018) (44) Prevención de la Obesidad Infantil: Un model de Base Comunitaria (POIBC) study from the Thao-Child Health Program (TCHP); Catalonia, Spain	Randomized cluster design Children (8–10 years old) living in four Catalan municipalities The communities were cities between 20,000 and 215,517 inhabitants	Community spaces and facilities 2 academic years (2012–2014) Baseline and 15 months follow-up	**Control** Baseline: 2 towns: *n* = 1,112 Follow-up: 2 towns: *n* = 1,112 **Intervention** Baseline: Two towns: *n* = 1,041 Follow-up: Two towns: *n* = 974	1) BMI sex-specific z-score: 0.078; *p* = 0.94 (adjusted by gender, age, lifestyle variables at baseline) and 0.012; *p* = 0.73 (adjusted by gender, age, maternal education, and baseline values of other indexes No statistically significant intervention effects in BMI z-score, but reductions in these outcomes were observed in both groups.
Ko et al. (2018) (31) (NOT COMPLETED) Together WE STRIDE; Lower Yakima Valley, Washington State, USA	Parallel quasi-experimental study Hispanic children (8–12 years old) attending elementary schools in the communities Rural communities	Families, schools, and community spaces and facilities Unknown NR	NR	NR
Swinburn et al. (2014) (39) Johnson et al. (2012) (71) Sanigorski et al. (2008) (72) Be Active Eat Well (BAEW); Colac, Victoria, Australia	Serial cross-sectional study (quasi-experimental non-randomized intervention study) Children (4–12 years old) and adults Colac (intervention community) is an urban community with good infrastructure and networks; the control community was rural	Families, schools, community spaces, and facilities 3 years (2003–2006) Baseline and 3 years post-intervention	**Control** Baseline: 1 community of 320,000 inhabitants (10 primary schools): *n* = 797 Follow-up: *n* = 621 **Intervention** Baseline: 1 town of 11,000 inhabitants (6 primary schools): *n* = 877 Follow-up: *n* = 660	NR (no values were reported for BMI, BMI z-score or prevalence overweight/obesity). No statistically significant differences between groups in BMI, BMI z-score, and prevalence of overweight/ obesity, but larger reductions were observed in the control area.
Bolton et al. (2017) (37) Health-Promoting Communities: Being Active Eating Well (HCP: BAEW); Victoria, Australia	Mixed methods and multilevel quasi-experimental randomized study. Cross-sectional (children and adolescents) and longitudinal (adults) designs Children (5–12 years old), children (12–18 years old), and adults in the workplace Secondary groups: older adults, carers, families, seniors, newly arrived migrants Rural and urban socioeconomically disadvantaged (according to SEIFA score) communities	Schools, workplaces, and community spaces and facilities 12 months to 2 years (2008/09–2010) Baseline and follow-up (12 months to 2 years)	**Control** Baseline: 18 primary schools, 15 secondary schools, seven workplaces Follow-up: 17 primary schools (*n* = 1,028), 14 secondary schools (*n* = 351), 7 workplaces **Intervention** Baseline: 5 communities: 18 primary schools, 15 secondary schools, seven workplaces Follow-up: 5 communities: 17 primary schools (*n* = 904), 14 secondary schools (*n* = 681), 7 workplaces (*n* = 135)	1) BMI z-score: −0.03 (Comm. 1), −0.03 (Comm. 2), 0.05 (Comm. 3), −0.29 (Comm. 4), NR (Comm. 5) Primary schools (Comm. 1 and 2): −0.02 Secondary schools: 0.01 Workplaces: NR All *p*-values> 0.05 except for Comm. 4. Adjusted for age, gender, level of disadvantage, baseline means, and clustering by school or workplace. 2) Prevalence of overweight/obesity: NR (values were not reported) Statistically significant differences for BMI z-score only for Community 4 (*p* < 0.05) in favor of the intervention, but not for the rest of the communities, or when all communities were pooled together.
Gittelsohn et al. (2017) (30) (not completed) OPREVENT 2; New Mexico, Wisconsin, USA	Stratified, group-randomized study design American Indian adults (18–75 years old) American Indian rural communities	Schools, worksites, and community spaces 12 months NR	NR	NR
Verbestel et al. (2015) (46) De Henauw et al. (2015) (58) Identification and prevention of lifestyle-induced health Effects in Children and infants (IDEFICS); 8 European countries (Belgium, Cyprus, Estonia, Germany, Hungary, Italy, Spain, and Sweden)	Non-randomized cluster-controlled trial using a before-and-after control group design. The study had two dimensions: an observational cohort (cross-sectional study) and an intervention study of a prevention program. Furthermore, they conducted three case–control studies with different sub-groups of children Preschool and school-aged children (2–10 years old), and their parents Not specified	Family, schools/kindergarten, and community spaces and facilities 2 years (2007/08–2009/10) Baseline and 2-year follow-up	**Control** Baseline: 1 area per country (8 to 35 schools per country): *n* = 7,746 Follow-up: *n* = 5,314 **Intervention** Baseline: 1 area per country (5 to 42 schools per country): *n* = 8,842 Follow-up: *n* = 5,727	No values were reported overall, only stratified by gender: 1) BMI z-score: Boys: −0.043 (*p* = 0.333) Girls: −0.095 (*p* = 0.042) 2) Prevalence of overweight/obesity (%): NR (values were not reported) Adjusted for age and SES of the parents, corrected for cluster design No statistically significant differences between groups for the prevalence of overweight/obesity with all schools pooled. Statistically significant lower increase in BMI z-score among girls in the intervention group compared to the control group.
Vinck et al. (2015) (47) VIASANO: French-speaking area of Belgium	Quasi-experimental study non-randomized Preschool children 3–4 years old and 5–6 years old, as well as the general public One urban and one rural town	Schools, community spaces, and facilities 3 years (2007–2010) Baseline and 3-year follow-up	**Control** The rest of the French-speaking area in Belgium Baseline: *n* = 76,864 Follow-up: *n* = 79,692 **Intervention** Baseline: 2 towns Follow-up: *n* = 1,484	1) Prevalence of overweight/obesity (%): NR (only the *p* value was reported: *p* = 0.058). No statistically significant differences between groups, but results were favorable for the intervention group (decrease in the prevalence of overweight/obesity observed).
Ayala et al. (2015) (29) (not completed) Our Choice/Nuestra Opción, Childhood Obesity Research Demonstration study; Imperial County and USA-Mexico border, California, USA	2 × 2 factorial study, quasi-experimental design Children (2–11 years old) and their parents Rural communities	Community spaces and facilities, health care and education centers, and schools 1 year (2011) NR	NR	NR
Crespo et al. (2012) (32) Aventuras para Niños Study; San Diego County, California, USA	Factorial design, randomized controlled community trial Latin children and their parents Schools with a majority of Latino enrollment (min. 70%)	Families, schools, and community areas 3 years Baseline (M1), 1-year post-intervention (M2), 1 year (M3) and 2 years (M4) follow-up	**Control** Baseline: 4 schools: *n* = 223 Follow-up: M1: *n* = 223; M2: *n* = 205; M3: *n* = 171; M4: *n* = 134 **Intervention** Baseline: 3 schools: *n* = 195 Follow-up: 3 schools: M2: *n* = 167; M3: *n* = 140; M4: *n* = 96	NR (no values were reported for BMI z-score, overweight prevalence, and obesity prevalence). No statistically significant changes in any of the weight measures. The greatest increase in overweight prevalence was observed for the Fam-only and Comm-only groups. The prevalence of obesity increased in all groups except in Fam-only. There were also no significant intervention effects on parents’ BMI or BMI category. Intervention effects did not vary after adding interaction terms (baseline weight status and child gender). All children increased their BMI z-score over the course of the study in all groups.
Pettman et al. (2014) (41) Eat Well Be Active (EWBA) Community Programs; South Australia	Quasi-experimental before-after interventional non-randomized study and repeat cross-sectional study. Mixed-methods evaluation framework. Children (0–18 years old) and their families Socioeconomically disadvantaged communities (outer-metropolitan suburbs and rural towns)	Child care and education centers, health, welfare, and indigenous agencies, community spaces, and facilities 3 years (2006–2009) Baseline and 3-year follow-up	**Control** Baseline: 2 communities (1 metropolitan, one rural): *n* = 541 Follow-up: 2 communities: *n* = 789 **Intervention** Baseline: 2 communities (1 metropolitan, one rural): *n* = 1,300 Follow-up: 2 communities: *n* = 1,005	NR (no values were reported for BMI z-score, prevalence of overweight, and prevalence of obesity) Statistically significant decrease in BMI z-score in both intervention and control groups for 4–5-year-olds, but these changes were not statistically significantly different between groups (*p* < 0.05). Larger decrease (statistically significant, *p* < 0.05) in overweight/obesity prevalence in the intervention compared to the control among 4–5-year-olds. No statistically significant changes in the outcomes for 10–12-year-olds, but a larger decrease in obesity prevalence in the intervention than in the comparator group.
Fotu et al. (2011) (51) Ma’alahi Youth Project (Pacific Obesity Prevention in Communities project – OPIC); Tonga and Vava’u, Pacific Islands	Quasi-experimental non-randomized study, longitudinal design Children/adolescents (11–19 years old) and their families Villages in different districts of the islands	School, community spaces, and facilities 3 years (2006–2008) Baseline and 3-year follow-up	**Control** Baseline: 6 schools: *n* = 1,396 Follow-up: 6 schools: *n* = 897 **Intervention** Baseline: 3 districts with 22 villages and seven schools: *n* = 1,083 Follow-up: *n* = 815	1) Age and gender-specific BMI z-score: Adjusted: −0.03 SE 0.03; *p* = 0.26 2) Adjusted pooled overweight/obesity prevalence (OR): −0.05 SE 0.24; *p* = 0.84 Adjusted for baseline variable, age at follow-up, height at follow-up, gender, and time between measurements. No statistically significant intervention effects in these outcomes.
Kremer et al. (2011) (52) The Healthy Youth Healthy Communities (part of the OPIC project), Fiji, Pacific Islands	Quasi-experimental non-randomized study Children (13–18 years old) Not specified	Schools, community spaces, and facilities 2/3 years (2005/06–2007/08) Baseline and 2-year follow-up	**Control** Baseline: 3 towns (11 secondary schools): *n* = 4,567 Follow-up: 3 towns (11 secondary schools): *n* = 2,069 **Intervention** Baseline: 1 community (7 schools): *n* = 2,670 Follow-up: 1 community (7 schools): *n* = 879	1) BMI z-score: 0.02 SE 0.02 (95% CI −0.02, 0.07; *p* = 0.33) 2) Proportion overweight/obesity prevalence: 0.34 SE 0.19 (95% CI −0.03, 0.71; *p* = 0.07) Adjusted for baseline measure, gender, ethnic subgroup, age and height at follow-up, duration between measures and clustering by school. No statistically significant differences between groups in these outcomes.
Lytvyak et al. (2016) (56) and Raine et al. (2013) (64) Healthy Alberta Communities (HAC); Alberta, Canada	Cross-sectional, non-randomized study Free-living adults (pregnant women and individuals in wheelchairs) from 18 years old Four communities in a geographically large, politically conservative, resource-rich province (rural towns and cities)	Community spaces and facilities 3 years (2006–2009) Baseline and 3-year follow-up	**Control** Baseline: National sample survey *n* = 3,725 Follow-up: *n* = 3,873 **Intervention** Baseline: 4 communities: *n* = 1,554 Follow-up: 4 communities: *n* = 1,808	1) BMI: NR (only the *p* value was reported: *p* > 0.05) 2) Prevalence of overweight/obesity: NR (only the *p* value was reported: *p* > 0.05) 3) NR (only the *p*-value was reported: *p* > 0.05) No statistically significant differences between groups in these outcomes.
Zhou et al. (2014) (53) A policy-driven multifaceted approach for early childhood physical fitness promotion; Beijing, China	Quasi-experimental before-and-after design with a comparison group, non-randomized study Children (3–5 years old) Not specified	Public childcare centers, community spaces, and facilities 12 months (2010/11) Baseline and 12-month follow-up	**Control** Baseline: 1 childcare center: *n* = 225 Follow-up: 1 childcare center: *n* = 218 **Intervention** Baseline: 1 childcare center: *n* = 148 Follow-up: 1 childcare center: *n* = 139	1) BMI: 0.19 (95% CI −0.06, 0.43; *p* > 0.05) 2) Age-specific BMI z-score: 0.15 (95% CI −0.01, 0.31; *p* > 0.05) Adjusted for baseline measures. No statistically significant differences between groups in these outcomes, but larger increase of BMI and BMI z-score in the intervention group.
De Silva-Sanigorski et al. (2010) (40) Romp & Chomp; Victoria, Australia	Repeat a cross-sectional, quasi-experimental, non-randomized controlled study Children (0–5 years old) and their families Large regional cities	Schools, care centers, maternal child health services, community health services, and regional immunization services 4 years (2004–2008) Baseline and 3-year follow-up	**Control** Baseline: 59 communities: *n* = 17,732 for 2-year-olds and *n* = 14,647 for 3.5-year-olds Follow-up: 59 communities: *n* = 21,911 for 2-year-olds and *n* = 19,050 for 3.5-year-olds **Intervention** Baseline: 2 communities: *n* = 1,587 for 2-year-olds and *n* = 1,194 for 3.5-year-olds Follow-up: 2 communities: *n* = 1,611 for 2-year-olds and *n* = 1,239 for 3.5-year-olds	NR (values were not reported for BMI, BMI z-score, and obesity prevalence) No statistically significant differences for BMI z-score, but a statistically significant shift in the distribution of weight status in the intervention sample, where a higher proportion of children were in the healthy-weight range in both age groups.
Taylor et al. (2007) (55) A Pilot Program for Lifestyle and Exercise (APPLE); Otago, New Zealand	Non-randomized controlled trial (pilot study) Children (5–12 years old) 2 semirural geographically separated communities	Schools and the community 2 years (2003–2005) Baseline, 1-year, and 2-year follow-up	**Control** Baseline: 3 schools: *n* = 219 children analyzed Follow-up: 3 schools: *n* = 217 analyzed at year 1 and 3 schools: *n* = 137 children analyzed at year 2 **Intervention** Baseline: 4 schools: *n* = 251 children analyzed Follow-up: 4 schools: *n* = 247 children analyzed at year 1 and 4 schools: *n* = 151 children analyzed at year 2.	1) BMI: NR (no values were reported) 2) BMI z-score: −0.09 (95% CI −0.18, −0.01) at year 1; −0.26 (95% CI −0.32, −0.21) at year 2(*p* values not reported) 3) Overweight prevalence (RR int. vs. control): 0.92 (95% CI 0.71, 1.18) at year 1; 0.88 (95% CI 0.69, 1.14) at year 2 (*p* values not reported) Statistically significant difference in BMI z-scores adjusted for baseline values, clustering, age, sex, activity rating, and television viewing. No statistically significant differences for overweight prevalence once adjusted for baseline values.
De Coen et al. (2012) (45) Prevention of Overweight among Pre-school and school children (POP); Flanders, Belgium	Cluster-randomized controlled trial Pre-primary and primary school children (3–6 years old) 2 low SES, two medium SES, and two high SES communities	Schools and the community 2 school years (2008–2010) Baseline and 2-year follow-up	**Control** Baseline: 3 communities (1 community low SES, one community medium SES, and one community high SES):13 schools (*n* = 2,416 children) Follow-up: 3 communities: 13 schools (*n* = 298 questionnaires analyzed, *n* = 442 height/weight measured) **Intervention** Baseline: 3 communities (1 community low SES, one community med SES, and one community high SES): 18 schools (*n* = 2034 children) Follow-up: 3 communities: 18 schools (*n* = 396 questionnaires analyzed, *n* = 670 weight/height measured)	1) BMI: NR (no values were reported) 2) BMI z-score: NR. Only reported for the low SES group: −0.46 statistical effect size with a statistical power of 0.80; *p* < 0.01 Intervention effect not statistically significant for the total sample, only for the low SES communities (*p* < 0.01).
Van de Kolk et al. (2020) (73) and Van de Kolk et al. (2019) (50) SuperFIT; Limburg, The Netherlands	Quasi-experimental research design Pre-school children (2–4 years old) and families Low SES urban neighborhoods	Schools, homes, and the community 1 year (2017–2018) Baseline, 3-month, and 1-year follow-ups	**Control** Baseline: 1 community (9 schools, *n* = 92 children) Follow-up: Not specified **Intervention** Baseline: 1 community (12 schools, *n* = 47 for full intervention including the family component and *n* = 52 for partial intervention excluding the family component) Follow-up: Not specified	1) BMI z-score: Control vs. full intervention: −0.09 (95% CI −0.31, 0.13; *p* = 0.44), ES = −0.09 (3 months) 0.00 (95% CI −0.25, 0.25; *p* = 0.99), ES = 0.01 (1 year) Control vs. partial intervention: 0.05 (95% CI −0.17, 026; *p* = 0.66), ES = 0.06 (3 months) −0.13 (95% CI −0.38, 011; *p* = 0.28), ES = −0.14 (1 year) No statistically significant differences for BMI z-score, with very small effect sizes between the full intervention or partial intervention and the control group in all follow-ups. BMI z-score improved significantly (*p* = 0.019) between baseline and follow-up within the partial intervention.
Strugnell et al. (2024) (42) and Strugnell et al. (2016) (74) Healthy Together Victoria (HTV), Australia	Cluster-randomized trial with repeated cross-sectional evaluations Secondary school children in grade 8 (13–15 years old) and grade 10 (15–16 years old), and adults Towns/suburbs in Victoria	Early childhood services, schools, workplaces, community, state, and local level 2 years (2014–2016) Baseline and 2-year follow-up	**Control** Baseline: 11 communities (13 schools *n* = 1,741 analyzed for BMI) Follow-up: 11 communities (23 schools *n* = 1,940 analyzed for BMI) N varies for each outcome **Intervention** Baseline: 12 communities (10 schools *n* = 1,139 analyzed for BMI) Follow-up: 12 communities (17 schools *n* = 1,394 analyzed for BMI) Schools were selected randomly and matched to intervention schools by sociodemographic index and prevalence of unhealthy weight among adults	1) BMI z-score: −0.07 (95% CI −0.23, 0.09; *p* = 0.382) 2) Overweight and obesity prevalence (%): −3.5 (95% CI −8.8, 1.8; *p* = 0.196) No statistically significant intervention effect in BMI and overweight/obesity prevalence, but the effects were favorable for the intervention group.
Van Dongen et al. (2022) (48) The Fit Lifestyle at School and at Home (FLASH); The Netherlands	Mixed methods, quasi-experimental design Secondary school children Not specified	Schools 3 years (2016–2019) Baseline and each year until the end of the study (2 to 3 measurements per cohort)	**Control** 4 schools (*n* = 460 for BMI) **Intervention** 4 schools (*n* = 460 for BMI)	1) BMI z-score: Baseline to 1st follow-up: −0.08 (95% CI −0.19, 0.03; *p* = 0.142) Baseline to 2nd follow-up: −0.09 (95% CI −0.21, 0.03; *p* = 0.155) Overall effect: −0.09 (95% CI −0.19, 0.02; *p* = 0.096) Adjusted for sex, educational level, and migration background. No statistically significant intervention effects for BMI z-score (small differences stable over time), but the effects were favorable for the intervention. When sample was stratified by cohorts (A, B and C based on the year of recruitment), intervention effects in BMI z-score were statistically significant for cohort B (2017).
Li et al. (2019) (54) and Li et al. (2017) (75) The Chinese Primary School Children Physical Activity and Dietary Behavior Changes Intervention (CHIRPY DRAGON); Guangzhou, China	Cluster-randomized controlled trial Children (6–7 years old) and families Urban districts	Schools and families 12 months Baseline and 1-year follow-up	**Control**Baseline: 20 schools (*n* = 826 measured) Follow-up: 20 schools (*n* = 769 analyzed) **Intervention**Baseline: 20 schools (*n* = 804 measured) Follow-up: 20 schools (*n* = 794 analyzed)	1) BMI z-score mean difference: −0.13 (95% CI −0.26, −0.00; *p* = 0.048) (baseline adjusted analysis); −0.13 (95% CI −0.26, −0.01; *p* = 0.041) (further adjusted analysis) 2) Overweight/obesity prevalence (OR): 0.53 (95% CI 0.27, 1.05; *p* = 0.067) (baseline adjusted analysis); 0.65 (95% CI 0.31, 1.36; *p* = 0.258) (further adjusted analysis) Adjusted for baseline outcome and school clustering (baseline); baseline outcome, prespecified school-level, child-level sociodemographic and behavioral, and measured sedentary time covariates (further analysis). Statistically significant favorable intervention effect on reducing BMI z-score, but not significant for overweight/obesity prevalence.
Natale et al. (2021) (36) Healthy Caregivers-Healthy Children (HC2); Miami, USA	Cluster-randomized controlled trial Children (18 months to 5 years old) and their parents Low-resource, ethnically diverse urban and rural communities	Child-care centers (CCCs), families 2 years for each phase (Phase 1: 2011–2013 and Phase 2: 2015–2017) Baseline and three follow-up timepoints (at 1, 1.5, and 2 years)	**Control** Phase 1: 16 centers (*n* = 457) Phase 2: 12 centers (*n* = 360) **Intervention** Phase 1: 12 centers (*n* = 767) Phase 2: 12 centers (*n* = 465)	1) PBMI Phase 1: 0.01, *p* = 0.002 (all weight groups); *p* = 0.7212 (healthy weight); *p* = 0.0007 (unhealthy weight) Phase 2: 0.16, *p* = 0.002 (all weight groups); *p* = 0.0042 (healthy weight); *p* = 0.2348 (unhealthy weight) No statistically significant changes over time. Statistically significant differences between control and intervention when weight groups are stratified. Mean child PBMI stayed healthy over the 2 years in both the control and intervention groups for phases 1 and 2 of the study.
Allender et al. (2021) (43) WHO STOPS Childhood Obesity? South West Victoria, Australia	Stepped-wedge cluster randomized trial Primary school children Not specified	Schools and the community 4 years (2015–2019) Baseline, 2-year, and 4-year follow-up	**Control** Baseline: 5 communities (25 schools, *n* = 972 students) Follow-up: 5 communities (25 schools, *n* = 1,041 at 2 years; 23 schools, *n* = 878 at 4 years) Baseline: 5 communities (15 schools, *n* = 820) Follow-up: 5 communities (23 schools, *n* = 1,370 students at 2 years; 21 schools, *n* = 1,259 at 4 years)	1) BMI z-score: 2-year follow-up: −0.09 (95% CI −0.24, 0.06; *p* = 0.217) 4-year follow-up: 0.10 (95% CI −0.06, 0.25; *p* = 0.219) 2) Overweight/obesity prevalence: 2-year follow-up: −2.5 (95% CI −7.2, 2.2; *p* = 0.297) 4-year follow-up: 4.5 (95% CI −0.5, 9.4; *p* = 0.079) Adjusted for school (clustering), group, wave (start year), interaction group x wave, school’s SES index, and type of school. No statistically significant intervention effect for BMI and obesity/overweight prevalence in the four years. Statistically significant interaction effect between trial arm and wave (time).
Buch-Andersen et al. (2021) (49) The Danish SoL Project, Bornholm (intervention) and Odsherred (controls), Denmark	Quasi-experimental study Children (3–8 years old) Low SES communities with a high prevalence of health risk factors	Childcare centers, primary schools, supermarkets, local mass media, and local communities 19 months (September 2012–April 2014) Baseline and 19-month follow-up	**Control** Baseline: 3 communities (4 childcare centers and three schools, *n* = 214 children) Follow-up: 3 communities (4 childcare centers and three schools, *n* = 175 children analyzed) **Intervention** Baseline: 3 communities (3 childcare centers and three schools, *n* = 238 children) Follow-up: 3 communities (3 childcare centers and three schools, *n* = 170 children analyzed)	1) BMI z-score: 0.19 (95% CI 0.08, 0.30; *p* = 0.0013) 2) Overweight prevalence: NR 3) Obesity prevalence: NR Adjusted for age, sex, parental education, household income, and family status. Statistically significant differences between intervention and control groups in favor of the control group (BMI z-scores decreased in the control group and increased in the intervention group).
French et al. (2023) (34) NET-Works; Minneapolis-Saint Paul, Minnesota, USA	Randomized controlled trial Children (2–4 years old at the time of inclusion) and their parents Low-income, racial/ethnic minority families	Primary care clinics, homes, and neighborhoods 66 months (2012–2018) Baseline, 12, 24, 36, and 66-month follow-ups	**Control**: Baseline: 12 primary care clinics (*n* = 265) Follow-up: 12 primary care clinics (*n* = 242 at 12 months, 224 at 24 months, 235 at 36 months, 151 at 66 months) **Intervention**: Baseline: 12 primary care clinics (*n* = 269 children) Follow-up: 12 primary clinics (*n* = 261 at 12 months, *n* = 257 at 24 months, *n* = 258 at 36 months, *n* = 187 at 66 months)	1) BMI (kg/m^2^): −0.19 (95% CI −0.64, 0.26) (36 months); −0.38 (95% CI −1.13, 0.37) (66 months) 2) BMI percentile: −0.33 (95% CI −3.93, 3.26) (36 months); 1.15 (95% CI −3.16, 5.45) (66 months) 3) BMI z-score: −0.07 (95% CI−0.23, 0.08) (36 months); −0.02 (95% CI −0.19, 0.15) (66 months) 4) Obesity prevalence (OR): 0.68 (95% CI 0.41, 1.14) (36 months); 0.71 (95% CI 0.43, 1.15) (66 months) All differences had a *p*-value> 0.05. All mean differences were adjusted for child, gender, and baseline value. No statistically significant effects at 66 months for BMI measures and obesity prevalence.
Gago et al. (2023) (35) Communities for Healthy Living (CHL) using Head Start programs (Greater Boston Head Start), Cambridge and Somerville, MA, USA	Stepped-wedge cluster randomized trial (all clusters received the intervention but started at different times) Children (33 months to 5 years old) and their families Low-income families	Families and Head Start Centers 3 years (but only 2 years of data included for evaluation due to the COVID-19 pandemic) Baseline (fall), follow-up (spring) in each intervention (academic) year	**Control**: 16 Head Start programs (*n* = 3,368 children enrolled, *n* = 2,579 children analyzed, *n* = 454 parents analyzed) **Intervention**: 10 Head Start programs (*n* = 1,631 children enrolled, *n* = 1,318 children analyzed, *n* = 501 parents analyzed) The same programs were in the intervention and control groups, but at different starting points	1) BMI z-score (only children): 0.06 (95% CI 0.02, 0.09; *p* < 0.01) unadjusted; 0.06 (95% CI 0.02, 0.10; *p* < 0.01) adjusted 2) Modified BMI z-score (adjusted version of BMI z-score), only children: 0.07 (95% CI 0.03, 0.10; *p* < 0.001) unadjusted; 0.07 (95% CI 0.03, 0.12; *p* < 0.01) adjusted Adjusted for parent race and ethnicity, educational level, and household employment status. There was a small statistically significant mean increase per year in BMI z-scores in the intervention compared to the control groups.

aOR – adjusted Odds Ratio, BMI – body mass index, BMI z-score – BMI standardized score, CDC – Center for Disease Control, CI – confidence interval, Comm. – community, Fam. – family, HRQoL – health-related quality of life, IOTF – International Obesity Task Force, IQR – interquartile range, NA – not applicable, NR – not reported, PBMI – body mass index percentile, RR – relative risk, SD – standard deviation, SE – standard error, SES – socioeconomic status, WC – waist circumference, WHO – World Health Organization, ys – years.

### 2.5 Risk of bias assessment

A risk of bias assessment (see Supplementary Table S5) of the eligible studies was performed independently by two reviewers (JS, ES), using the risk of bias assessment tool developed by the Cochrane Collaboration for non-randomized studies (22). The bias assessment was done for interventions at both the individual and cluster levels.

## 3 Results

### 3.1 Search results

A total of 2,724 publications were retrieved from the database search, and eight additional publications were identified from a previous systematic review, of which 33 met our inclusion criteria (see PRISMA Diagram in the Supplementary Figure S1). We searched for additional publications of the studies; however, only the study protocol was identified, and four relevant articles were added. The most frequent reasons for exclusion were that interventions did not comply with the definition of CBI, there was no control group for comparison, the intervention period was less than 12 months, or that none of the outcomes of our interest (i.e., quantitative weight-related outcomes) were measured. Since some publications referred to the same intervention program, they were grouped into the same study, leading to a total of 28 studies included in this review.

A total of three studies out of the 28 identified were not yet completed. Our reviewers reached out to the authors for these studies, but did not get a response. Therefore, for these studies, only the study characteristics were extracted (29–31).

A summary of the study characteristics and results is provided in Table 1. More detailed information about the studies and health outcomes can be found in the Supplementary Tables S3, S4.

### 3.2 Study characteristics

Regarding study design, a total of 17 studies were non-randomized controlled trials or cross-sectional studies, and 11 studies were cluster-randomized controlled trials. The studies were conducted mostly in the USA (*n* = 8) (29–36), Australia (*n* = 7) (37–43), and European countries (*n* = 7) (44–50), followed by Pacific Islands (*n* = 2) (51, 52), China (*n* = 2) (53, 54), New Zealand (*n* = 1) (55), and Canada (*n* = 1) (56). The target populations of the interventions were children only (*n* = 12), children and their parents or families (*n* = 9), children and adults (*n* = 5), or adults only (*n* = 2). Intervention communities ranged in size from 45 to 5,800 people, whereas the control communities ranged from 90 to almost 77,000 individuals. All interventions addressed both physical activity and nutrition behavior. They were based on various theoretical frameworks, including socioecological models, capacity-building approaches, social cognitive theory, the health belief model, community-based participatory research (CBPR), the “Ensemble Prévenons l’Obésité Des Enfants” (EPODE – Together Let us Prevent Childhood Obesity) approach, and the theory of change. The duration of the intervention ranged between one and five years, and the implementation settings included community spaces, such as gardens, restaurants, shops, schools, health and care centers, workplaces, and households. Schools were the most frequent setting for implementation, being included in a total of 21 studies. The main components of the interventions included stakeholder engagement and community involvement, social marketing, educational events, physical activity events, changes in the built environment, and policy changes. The same core components were integrated into all interventions, but to a different extent and adapted to the context. The most frequently reported outcome measures were BMI and BMI z-score, which were assessed in all included studies. The prevalence of overweight and obesity was evaluated in a total of 18 studies.

### 3.3 Intervention effects

The quantitative effect of the intervention compared to the control group was calculated and reported for at least one of the primary outcomes in 18 studies (32–36, 38, 42–45, 48–55). In the remaining studies, the effect size (intervention vs. control) was neither calculated nor reported for any primary outcome.

#### 3.3.1 BMI measures

BMI was assessed in included studies using at least one of the following measures: BMI, PBMI, or BMI z-score in all included studies, with the intervention effect being calculated and reported numerically for the total study population in 16 studies (33–36, 38, 42–44, 48–55).

In three other studies (47, 56, 57), the effect was not reported, and only the statistical significance as a *p-*value (*p* > 0.05 in all three studies) was provided.

Among the 16 studies for which the intervention effect of the total study population was calculated, a favorable effect for the intervention, with BMI z-score mean differences ranging from −0.26 to −0.03, was observed in eleven studies in at least one follow-up (33, 34, 36, 38, 42, 43, 48, 50, 51, 54, 55), indicating a lower increase in the BMI z-score from baseline to follow-up or a larger decrease in the BMI z-score in the intervention group. These effects were statistically significant in four out of the eleven studies. The largest effects were observed in the APPLE Project by Taylor et al. (55) at two-year follow-up; CHIRPY DRAGON by Li et al. (54) at one-year follow-up and the Shape Up Somerville, as reported by Coffield et al. (33) at one-year follow-up, with mean differences of −0.26 (95% CI −0.32 to −0.21), −0.13 (95% CI −0.26 to 0.01; *p* = 0.041), and −0.1005 (95% CI −0.1541 to −0.0555; *p* = 0.001), respectively.

In the remaining seven out of these eleven studies, the reported effects were not statistically significant. Despite the intervention effect being favorable for BMI z-scores, in three of these studies (38, 42, 51), the BMI z-score increased from baseline to follow-up in both study groups (intervention and control arms), but this increase was lower in the intervention arm. One of these studies (43) had a favorable intervention effect at the two-year follow-up, but not at the final follow-up (at four years). In the study by van de Kolk et al. (50), the effects were only favorable at three months for the full intervention, but not for the partial intervention.

In five out of the 16 studies (35, 44, 49, 52, 53), outcomes were favorable for the control group, with a greater reduction or smaller increase in BMI z-score over time compared to the intervention group. Effect sizes ranged from 0.19 to 0.02, with only two studies (35, 49) reporting statistically significant differences. Moreover, Swinburn et al. (39) reported greater BMI z-score reductions in the control community compared to the intervention, but the magnitude of the effect and statistical significance were not calculated.

Three other studies reported intervention effects for a certain group or community of the study population. De Coen et al. (45) reported results only for the low socioeconomic status (SES) community, with a statistically significant intervention effect of −0.46 (*p* < 0.01), where the BMI z-score decreased in the intervention group and increased in the control arm from baseline to follow-up. Verbestel et al. (46) and De Henauw et al. (58) (IDEFICS study) reported the intervention effects only stratified by gender. A statistically significant (*p* = 0.042) lower increase in BMI z-score was observed among girls in the intervention group compared to the control group. Bolton et al. (37) showed that the effects were reported by the community, with one showing a statistically significant difference in BMI z-score in favor of the intervention (−0.29; *p* < 0.05), but not for the four other evaluated communities.

#### 3.3.2 Prevalence of overweight and obesity

Among the 18 studies reporting on overweight or obesity outcomes, nine studies assessed the prevalence of overweight (32, 38, 40, 41, 49, 51, 52, 55, 56), nine studies assessed the prevalence of obesity (32, 34, 38, 40, 41, 49, 51, 52, 56), and eight studies assessed the pooled prevalences of overweight and obesity (33, 37, 39, 42, 43, 46, 47, 54).

Only nine studies presented the effect of the intervention (33, 34, 38, 42, 43, 51, 52, 54, 55). In seven of these nine studies, the intervention effect was beneficial at all follow-ups. However, the effect was not statistically significant in any of these studies after controlling for potential confounding factors, except in one study by Coffield et al. (33) (Shape Up Somerville), which achieved an adjusted OR of 0.71 (intervention vs. control, *p* = 0.004).

Among the studies that showed a beneficial intervention effect but which were not statistically significant, Bell et al. (38) demonstrated a 23% lower likelihood of children being overweight and a 49% lower likelihood of children being obese at the final follow-up, thanks to the intervention. Li et al. (54) also showed lower odds of being overweight/obese in the intervention schools compared to the control schools with an adjusted OR of 0.65 (95% CI 0.31, 1.36; *p* = 0.258). Similarly, a favorable intervention effect was also achieved in the study by French et al. (34), which reported an OR of 0.71 (95% CI 0.41, 1.14; *p* > 0.05). Taylor et al. (55) reported a slightly lower overweight likelihood in the intervention arm than in the control arm with a relative risk of 0.88 (95% CI 0.69, 1.14). Strugnell et al. (42) reported a −3.5% (95% CI −8.8, 1.8, *p* = 0.196) difference in change of overweight and obesity prevalence, indicating a lower prevalence in the intervention group. Fotu et al. (51) observed a smaller increase in overweight and obesity prevalence in the intervention compared to the control, with an OR of −0.05 (SE 0.24; *p* = 0.84). Allender et al. (43) reported the adjusted difference in change of overweight and obesity prevalence between intervention and control groups as −2.5% (95% CI −7.2, 2.2, *p* = 0.297) at two years and 4.5% (95% CI −0.5, 9.4, *p* = 0.079) at four years, indicating a greater increase in prevalence in the intervention group over time. Finally, Kremer et al. (52) reported a larger proportion of overweight/obese in the intervention group at follow-up relative to the comparison group, with a difference of 0.34 (SE 0.19, 95% CI −0.03, 0.71; *p* = 0.07).

Where the effect size was not quantified or reported, the studies presented separate outcomes for the intervention and control groups without comparison measures between groups. For these studies, we found a variety of outcomes. We observed a small increase (less than 5%) in the prevalence of overweight or obesity in the intervention community as reported by De Henauw et al. (58) and Crespo et al. (32). However, this increase was smaller than in the control community. In the study by Buch-Andersen (49), there was a small increase (less than 2%) in both the prevalence of overweight and obesity in the intervention and control groups. Five studies, including Vinck et al. (47), Swinburn et al. (39), Pettman et al. (41), Lytvyak et al. (56), and De Silva-Sanigorski (40), reported a decrease in the prevalence of overweight and obesity in the intervention group. However, in Swinburn et al. (39) and Lytvyak et al. (56), the decrease in overweight prevalence was larger in the control group. Obesity rates rose more in the control group in Lytvyak et al. (56). Finally, although Bolton et al. (37) measured the prevalence of overweight and obesity, they did not report baseline values; hence, the pre-post intervention difference could not be calculated.

### 3.4 Risk of bias

The assessment of the risk of bias for all included studies is shown in Supplementary Table S5. The overall risk of bias was moderate to high. All studies were subject to selection bias, as communities were chosen based on community capacities and location differences. In some cases, the samples taken at baseline and follow-up were not the same due to the high volume of dropouts and low participation rates during follow-up, which can lead to attrition bias. Most studies adjusted for confounders; however, the methods for confounder selection were not reported.

## 4 Discussion

This review aimed to update previous evidence on CBIs for obesity prevention in children, adolescents, and adults. We included 37 publications representing 28 distinct projects. This systematic review updated an earlier published review (20) on CBIs to prevent obesity in children, adolescents, and adults.

Our review found limited evidence on whether CBIs are effective in reducing overweight and obesity. Across studies, reported intervention effects on preventing overweight and obesity were small and varied, ranging from unfavorable to no effects to favorable intervention effects. Nevertheless, most studies showed more favorable outcomes in the intervention communities compared to the control communities in both assessed primary outcomes. The greatest effect in the reduction of BMI z-score was achieved in the study by Li et al. (54) (CHIRPY DRAGON Project, China), followed by the study by Taylor et al. (55) (APPLE Project, New Zealand) and the study by Coffield et al. (33) (Shape Up Somerville, United States) over a follow-up period of one to two years. In particular, Li et al. (54) reported a BMI z-score mean difference of −0.13 (95% CI −0.26 to −0.01) in the adjusted analysis, which was statistically significant. Common features of these three studies were that they targeted primary school children (6 to 12 years old) and aimed to promote healthy eating and physical activity tailored to their communities. All three interventions also had a strong emphasis on the school setting and involved parents and families in their programs. These features may have been key contributors to their effectiveness, as previous systematic reviews have highlighted the beneficial effects of school-based programs with home involvement (21), particularly for this age group (10). About the prevalence of overweight and obesity, one study by Coffield et al. (33) (Shape Up Somerville) showed a statistically significant difference in favor of the intervention.

Our review targeted the general population and excluded publications that focused on a specific population subgroup. Population-based strategies may have a differential effect on disadvantaged groups, whose choices are strongly shaped by the environment (59). Thus, it might be valuable to conduct subgroup analysis by SES or ethnicity to examine differences in effects and identify communities that would benefit the most from CBIs. In fact, the study by De Coen et al. (45) showed statistically significant, favorable intervention effects for BMI z-score when examining the community with a low SES alone.

Overall, despite the modest or uncertain effects identified in our review, there is growing support for the implementation of CBIs to prevent overweight and obesity. The general trend of the included studies had favorable outcomes in the intervention communities. Previous meta-analyses pooling the effects of several obesity prevention interventions in childhood have demonstrated modest but favorable effects (10, 25). Some CBIs for obesity prevention have shown larger effects than others (60), and it is, therefore, essential to identify the specific aspects that are key to the success of CBIs to ensure their efficacy. For instance, a strong school component has been identified as a common factor of successful interventions (21). Community capacity, readiness, and engagement in addressing change are also key factors in ensuring that interventions are sustainable and capable of achieving change (19). Considering that the environmental changes in the last decades have played an important role in the increase of the overweight and obesity prevalence rates (5) and the larger population reach of CBIs (8, 12, 14), CBIs that modify environmental drivers of obesity are more likely to be cost-effective compared to individually tailored prevention strategies in the healthcare settings (61, 62). CBIs focus on prevention rather than treatment, thereby reducing the long-term healthcare expenditures associated with obesity-related conditions. By targeting entire populations, CBIs can achieve a broad reach and, consequently, improve BMI not only in the very critical obese subpopulations but also shift the entire BMI distribution to a more favorable one. Even modest shifts in behavior or weight can translate into substantial cumulative health and economic benefits at the population level. Furthermore, such population-based community interventions often utilize existing infrastructures, such as schools, local organizations, and public spaces, which minimizes implementation costs and increases cost-effectiveness. Finally, CBIs are more effective in reducing socioeconomic inequalities because they can engage inaccessible populations (14, 19). Although the current available evidence is narrow, published cost-effectiveness analyses (CEAs) for obesity prevention have mostly reported cost-effective results (61).

The small or no effects observed in our review might be partially explained by the methodological challenges in measuring and capturing the true effects of these interventions. The BMI at baseline might have an impact on the outcomes, whereby an effect might be easier to find in populations with a higher baseline BMI. Furthermore, the follow-up time in these studies (up to five years) may not be long enough to capture potential effects that might appear later in time. Moreover, there might be a spillover effect from the people who were part of the intervention to other generations and family members, but such effects are difficult to detect and evaluate. The diversity of components (physical activity and nutrition policies, health education trainings, different settings, social marketing, infrastructure changes, physical activity trainings, among others) included in the interventions also poses difficulties in identifying which elements are essential for successful interventions (14, 63). Lastly, the comparability of the studies is hindered by the heterogeneity of study designs, outcome measurements, data collection methods, and follow-up timepoints.

Previous systematic reviews have predominantly focused on interventions targeting children. In our review, we identified two studies targeting and evaluating outcomes only in adults. One of these studies (30) was not completed, and its results are therefore unavailable. The other study (64) did not report effect sizes for any of the outcomes. However, the authors stated that there were no statistically significant differences between the control and the intervention groups. Together, these two studies do not allow for drawing reliable conclusions about the effectiveness of CBIs in adults, thereby highlighting the research gap previously mentioned by Wolfenden et al. (20) regarding the limited evidence available for this population. The rationale behind this gap might be that CBIs targeting children can utilize schools as a universal setting to base the intervention. However, in adulthood, equivalent settings are more difficult to find (61). Furthermore, existing evidence has shown a strong association between childhood and adult obesity; obesity in childhood and, especially in adolescence, is very likely (over five-fold likelihood) to persist in adulthood, and it is difficult to reverse (63, 65). Thus, intervening at an early age, when nutrition and physical activity habits are formed, is crucial to slow down the growing trend of childhood and adolescent obesity and, in turn, prevent the multiple obesity-related co-morbidities at these ages (63, 65). However, it is important to note that most of the overall burden of obesity in adulthood is not primarily determined by childhood obesity, and the largest proportion of obesity has a later onset (65). In fact, 70% of adults with obesity were not obese in childhood or adolescence (65, 66). This highlights the importance of CBIs that target both children and adults to achieve a larger impact on reducing the overall obesity burden. Targeting solely children with obesity and overweight is hardly going to have a large impact in reducing the prevalence of obesity in adulthood (63).

We found that most CBIs for obesity prevention included in our analysis were implemented in highly developed (high-income) countries, with far fewer conducted in developing (middle-income) countries. The limited implementation of CBIs in these settings (12) likely reflects the additional challenges faced by those countries, including competing public health priorities that often divert attention and resources away from non-communicable disease prevention (67). Budget constraints, limited infrastructure, and shortages of trained personnel further hinder both implementation and sustainability of such programs, potentially undermining their effectiveness (68). Addressing these barriers and limitations will require stronger international collaboration, greater community engagement, and innovative approaches tailored to the realities of low- and middle-income countries (LMICs) (68).

The risk of bias of the studies was moderate to high owing to various reasons, which is consistent with other systematic reviews (10, 16, 63). In most of the studies, communities were not randomized, and they were selected based on the capacity and existing infrastructure, which may result in confounding bias if no adjustment for confounders is performed (8, 19). Second, the high number of lost-to-follow-up gives rise to attrition bias. Therefore, results should be considered only as an indication, given the suboptimal methodological quality of the studies. The low strength and quality of the evidence might reflect the natural characteristics of CBIs: it is complex to carry out RCTs because it is challenging to isolate intervention effects from other influential factors or find appropriate matching control communities. Studies are also prone to high dropout rates and contamination between communities.

Our systematic review has several limitations. Firstly, regarding the review criteria, we only focused on BMI and BMI-related measures, while there might also be positive effects reflected by other outcomes, such as behavioral outcomes (dietary and physical activity habits) or knowledge on healthy nutrition, which are an excellent baseline to prevent overweight and obesity. Thus, BMI alone might not be representative of all the potential benefits of interventions. Secondly, BMI has its own flaws: it does not distinguish between muscle tissue and fat, ignores body fat distribution, and does not consider ethnic, age, or gender variations. Following on this, not including other weight-related measures such as waist circumference or body fat percentage poses a methodological limitation in the study, as such measures are often regarded as more accurate indicators of overall health. Incorporating these additional measures would more accurately capture health risks associated with factors like abdominal fat and yield a clearer picture of the population’s health status. Despite its limitations, we chose BMI because it remains the most widely used measure of overweight and obesity worldwide, valued for its ease of assessment and feasibility for monitoring weight over time. Moreover, the scope of this review was limited to overweight and obesity. Thirdly, as our systematic review was limited to a single database, some relevant publications might have been missed in our search. Finally, due to the high heterogeneity of the studies included in this review, we were unable to pool intervention effects in a meta-analysis. Instead, we relied on a systematic and narrative synthesis of the evidence. Contributing factors included the lack of or limited reporting of effects adjusted for confounding, as well as considerable heterogeneity in study designs, study populations, and intervention types. A potential next step to provide quantitative evidence on this subject could be the performance of subgroup analyses stratifying for relevant factors such as intervention setting, outcomes, or target population.

We argue that a consensus on the study design, evaluation, and reporting of CBIs is required to facilitate comparability of studies and help identify the components of interventions that work best for children and the general adult population. Future studies should drive efforts towards this direction. Furthermore, although cost-effectiveness analyses of CBIs are currently limited (9), they have the potential to serve as a valuable tool for supporting decision-making processes. Future research should also focus on evaluating the cost-effectiveness of various interventions to prevent overweight and obesity in different country settings.

## 5 Conclusion

Most of the CBIs included in this review reporting the effect size of at least one primary outcome showed a positive but relatively small effect in reducing obesity and overweight among children. However, a minority of studies showed better outcomes achieved in the control group than in the intervention group. Most of the interventions targeted children, with few studies focusing on the adult population. Overall, our review found insufficient evidence to draw firm conclusions about the effectiveness of CBIs in preventing overweight and obesity. While creating health-promoting environments can influence population-wide nutrition and physical activity behaviors by making healthy choices more accessible, methodological challenges make it difficult to accurately capture the true effects of CBIs. Research efforts in this field should explore long-term effects of CBIs to prevent overweight and obesity, strengthen the focus on the adult population, and compare the effectiveness of CBIs among different population subgroups.

## Data Availability

The original contributions presented in the study are included in the article/Supplementary material, further inquiries can be directed to the corresponding author.
