# Supplementary material for: Systematic review on community-based interventions targeting prevention of overweight and obesity in children and adolescents
Source: Front Public Health. 2025 Oct 29;13:1687963. doi: 10.3389/fpubh.2025.1687963 (PMC12605465; doi:10.3389/fpubh.2025.1687963)
Supplement: Supplementary file 1 [file Table_1.docx]

Supplementary Material

**Table of contents**

[Figure S1. PRISMA diagram. 2](#_Toc203033813)

[Table S1. Inclusion and exclusion criteria of the systematic review of CBIs. 3](#_Toc203033814)

[Table S2. Search strategy for PubMed for the systematic review of CBIs. 4](#_Toc203033815)

[Table S3. Study and community-based intervention characteristics. 5](#_Toc203033816)

[Table S4. Overview of results of the included studies. 19](#_Toc203033817)

[Table S5. Risk of bias assessment of included studies. 35](#_Toc203033818)

[References 38](#_Toc203033819)

Figure S1. PRISMA diagram.


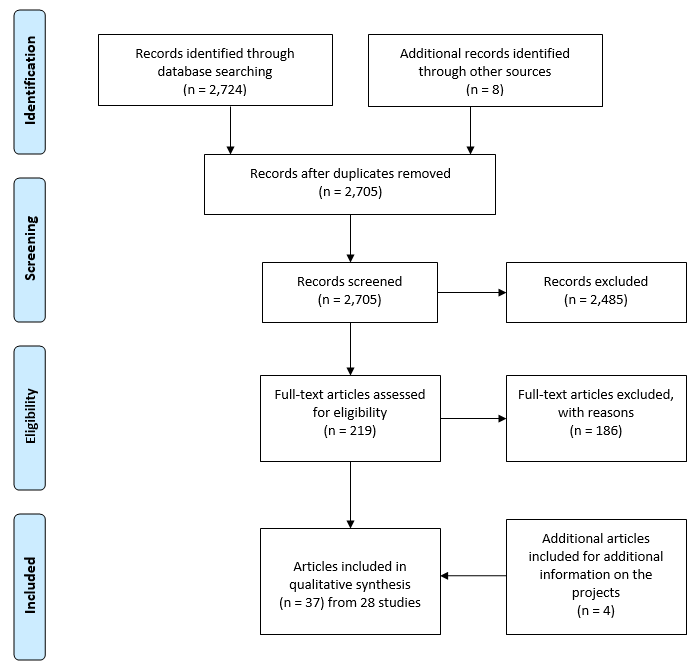


Table S1. Inclusion and exclusion criteria of the systematic review of CBIs.

| **Parameters** | **Inclusion** | **Exclusion** |
| --- | --- | --- |
| **Population** | General population representative of a community, who are free living and defined along geographical boundaries from middle and high-income countries. | Sub-groups of the population who are isolated from a community, high-risk individuals (obese and overweight only or with a medical condition). |
| **Intervention** | - Targeting a community to change physical activity and/or nutrition to prevent obesity implemented in urban or moderately rural areas - Multi-component - Multi-setting - Involvement of community stakeholders - Minimum 12 months of implementation   Blackburn: community-based prevention programs are integrated and comprehensive, not limited to medical care settings, and systematically involve community leaders, social networks, mass communication campaigns, and direct education of the general population. | - Only implemented in one setting - Interventions aimed at treating obesity or overweight - Interventions implemented in remote areas - Preventing obesity is a secondary aim - Physical activity and nutrition are secondary interventions |
| **Comparators** | A community without the intervention (nothing) or “standard of care”, or compare before-after intervention | No comparator/control |
| **Outcomes** | Quantitative weight-related outcomes: BMI, BMI z-score*, obesity/overweight prevalence | All others |
| **Study designs** | RCTs, non-RCTs, cluster-controlled trials, quasi-experimental studies | Observational studies, systematic reviews, meta-analysis, natural experiments, |
| **Time limits** | January 2011 (end of last systematic review) – September 2024 | Outside of the timeframe |
| **Languages** | English, Spanish, Catalan, German | All others |

**BMI adjusted for age and gender.*

Abbreviations: *BMI – body mass index, BMI z-score – standardized BMI, RCT – randomized controlled trial*

Table S2. Search strategy for PubMed for the systematic review of CBIs.

| **N** | **Category** | **Terms** | **Results** |
| --- | --- | --- | --- |
| **1** | **Disease: obesity** | (((((((((obese[Title/Abstract]) OR overweight[Title/Abstract]) OR "over weight"[Title/Abstract]) OR adiposity[Title/Abstract]) OR (body[Title/Abstract] AND fat[Title/Abstract])) OR (unhealthy[Title/Abstract] AND weight[Title/Abstract])) OR overnutrition[Title/Abstract]) OR "over nutrition"[Title/Abstract]) OR (high[Title/Abstract] AND BMI[Title/Abstract])) OR obesity[Title/Abstract] | 568,216 |
| **2** | **Prevention** | (((((health AND (promote OR promotion))) OR (obesity/Prevention and control[MeSH Terms])) OR prevent*) OR (Prevention[MeSH Subheading] AND control[MeSH Subheading])) OR (health AND education) | 4,645,132 |
| **3** | **Community** | ("community health services"[MeSH Terms] OR "community health planning"[MeSH Terms] OR "multi-level"[All Fields] OR "multi-component"[All Fields] OR "community-level"[All Fields] OR "multi-setting"[All Fields] OR "whole-of-systems"[All Fields] OR "whole-system"[All Fields] OR "community-based"[All Fields] OR "community-based participatory research"[MeSH Terms] OR "community-based participatory research"[All Fields] OR "community participation"[All Fields] OR "multi-sectorial"[All Fields] OR "multi-sector"[All Fields] OR "multi-faceted"[All Fields] OR "multifaceted"[All Fields] OR "multi-disciplinary"[All Fields]) | 522,551 |
| **4** | **Intervention** | (((((((((((strategy) OR intervention) OR program) OR initiative) OR policy) OR action) OR measure) OR approach) OR therapy) OR management) OR project) OR health planning[MeSH Terms] | 23,797,428 |
| **5** | **Outcomes** | Body-mass-index OR BMI OR "body weight" OR (weight AND (change OR gain OR loss OR reduction)) OR "skinfold thickness" OR "waist circumference" OR waist-to-height ratio OR (anthropometr* AND (measure OR measurements OR parameters OR indicators OR assessment OR profile OR characteristics)) OR "fat reduction" | 1,257,656 |
| **6** | **All** | 1 AND 2 AND 3 AND 4 AND 5 | 7,414 |
| **7** | **Filters** | 6 AND Filter: 01/01/2011 – 31/12/2024, (full-text availability) | 2,724 |

Table S3. Study and community-based intervention characteristics.

| **Authors, Years** | **Study name, country** | **Aim** | **Study design and target population and communities** | **Health outcomes** | **Theoretical framework** | **Duration of intervention, settings** | **Intervention description and components** |
| --- | --- | --- | --- | --- | --- | --- | --- |
| **Coffield et al. 2015 (1)**  **Economos et al. 2007 (2)**  **Economos et al. 2013 (3)** | Shape Up Somerville (SUS); Massachusetts, USA | Investigate whether SUS can prevent weight gain in young children and can influence the BMI of parents whose children attended the schools involved in the SUS intervention | Quasi-experimental, non-randomized controlled trial  Children grade 1-3 in elementary schools; parents of children attending SUS schools | Anthropometric outcomes (BMI z-score, self-reported BMI change from baseline, other anthropometric information), household sociodemographics, familiarity and exposure to SUS, SUS-induced behavioral changes | Community-based participatory research (CBPR) | 3 years (2002 – 2004/05)  Elementary schools, community spaces and facilities, homes | PA and diet; The intervention was developed to influence every part of an early elementary schoolchild´s day in order to increase availability of healthy foods and PA opportunities. They partnered with community members of three culturally diverse communities. Schools received a financial incentive for participation. It involved extensive outcome and process evaluation, documented participation and adherences. |
| **Bell et al. 2019 (4)** | Obesity Prevention and Lifestyle (OPAL); South Australia | Determine the effectiveness of a South Australian community-based, multi-setting, multi-strategy intervention in increasing healthy weight prevalence of the primary school children from phase 1 and 2 OPAL communities | Quasi-experimental repeated cross-sectional design, non-randomized  Children 4-5 years old, 9-11 years old, 14-16 years old  Disadvantaged communities (low SES according to the Index of Relative Socio-economic Disadvantage, IRSD) | Primary outcomes: Anthropometric (weight status and BMI), healthy weight prevalence, HRQoL; Secondary outcomes: Behavior outcomes | Social marketing, community development and socioecological systems theory. Community capacity building (CCB) approach. | 2-3 years  Community spaces and facilities, workplaces, schools | PA and diet; The intervention was shaped in each community depending on their needs. It included six marketing themes related to promote healthy eating and physical activity. It is based on EPODE*, a successful intervention from France, but OPAL targeted a wider age range (children aged 0-12) years and was implemented in both regional and metropolitan communities. There was a central coordination unit working together with local community teams. |
| **Gomez et al. 2018 (5)** | Prevención de la Obesidad Infantil: Un model de Base Comunitaria (POIBC) study from the Thao-Child Health Program (TCHP); Catalonia, Spain | Determine the efficacy of the TCHP program on BMI and waist-to-height ratio (WHtR) as the main outcome, and dietary quality and PA as the secondary outcome, in children from four selected Catalan municipalities | Randomized cluster design (municipalities are the unit)  Children 8-10 years old living in four Catalan municipalities  The communities were cities between 20,000 and 215,517 inhabitants | Anthropometric outcomes (weight, height, BMI, BMI z-score, WC, WHtR), incidence of obesity and abdominal obesity, behavioral outcomes (physical activity level, adherence to the Mediterranean diet) | Attitude-social influence-self efficacy model, social marketing strategies for public health and CBI guidelines for obesity prevention | 2 academic years (2012-2014)  Community spaces and facilities | PA and diet; The intervention was led by the city council with a local coordinator, who received training and ongoing support. The intervention was composed of several activities, including workshops, events, trainings, media promotion and information materials distribution. An annual evaluation protocol for each town was carried out too. |
| **Ko et al. 2018 (6) (NOT COMPLETED)** | Together WE STRIDE; Lower Yakima Valley, Washington State, USA | Study the effectiveness of the comprehensive, community-wide multi-level intervention at reducing BMI z-scores, dietary intake and PA among Hispanic children living in a rural area | Parallel quasi-experimental study  Hispanic children (8-12 years old) attending elementary schools in the communities  Rural communities | Primary outcomes: Anthropometric measures (height, weight, BMI z-scores)  Secondary outcomes: Diet and PA behavior | CBPR following the 9 core principles; social ecological framework | Unknown  Families, schools and community spaces and facilities | PA and diet; Different community advisory boards (CABs) consisting of diverse groups of stakeholders and in partnership with researchers developed and implemented the intervention, which included strategies at a family, school and community level. |
| **Swinburn et al. 2014 (7)**  **Johnson et al. 2012 (8)**  **Sanigorski et al. 2008 (9)** | Be Active Eat Well (BAEW); Colac, Victoria, Australia | Measure the impact of BAEW 3 years after the intervention finished | Serial cross-sectional study (quasi-experimental non-randomized intervention study)  Children (4-12 years old) and adults  Colac (intervention community), is an urban community with good infrastructure and networks; the control community was rural | Anthropometric measures (height, weight, BMI z-score), prevalence of overweight and obesity, lunch box audits, diet and PA behaviors and economic investment in obesity prevention | Community capacity-building principles | 3 years (2003-2006)  Families, schools, community spaces and facilities | PA and diet; It is an obesity prevention program to reduce unhealthy weight gain in children. The intervention was designed, planned and implemented by the key organizations and other stakeholders in Colac. It had 10 objectives related to capacity building, increasing awareness of the project, evidence-based behavior changes, parental education and support. |
| **Bolton et al. 2017 (10)** | Health-Promoting Communities: Being Active Eating Well (HCP:BAEW); Victoria, Australia | Evaluate the impact of HCP: BAEW initiative in increasing healthy eating and PA adapted to the community context in five separate communities in order to expand CB obesity prevention efforts in Victoria and provide recommendations for future interventions | Mixed methods and multilevel quasi-experimental randomized study. Cross-sectional (children and adolescents) and longitudinal (adults) designs  Children 5-12 years old, children 12-18 years old and adults in the workplace  Secondary groups: older adults, carers, families, seniors, newly arrived migrants  Rural and urban socioeconomically disadvantaged (according to SEIFA score) communities | Primary outcomes: anthropometric measures (weight, waist, BMI and BMI z-score)  Other outcomes:  Obesity-related behavior, environmental data, community context and implementation factors, quality-of-life (QoL) | - | 12 months to 2 years (2008/09-2010)  Schools, workplaces and community spaces and facilities | PA and diet; The project objectives were developed using the Analysis Grid for Environments Linked to Obesity framework, and involving stakeholders, expert advisory groups, the project board, evaluators as well as reviewing literature. The intervention focused on social marketing, stakeholder engagement, network and partnership development, capacity building, community strengthening, needs assessment, policy development and implementation. |
| **Gittelsohn et al. 2017 (11)**  **(NOT COMPLETED)** | OPREVENT 2; New Mexico, Wisconsin, USA | Reduce and prevent adult obesity in five America Indian communities | Stratified, group randomized study design  American Indian adults (18-75 years old)  American Indian communities (rural) | Anthropometric measures (weight, BMI, blood pressure, waist and hip circumference, % body fat), nutrition and PA behaviors, exposure to specific components and materials (dose received), process evaluation | Bandura's social cognitive theory and Bronfenbrenner's social ecological model | 12 months in total  Schools, worksites and community spaces | PA and diet; It was a multi-institutional intervention. One of its key elements was to ensure the sustainability of the program by having policy level monthly meetings with the objective to form relationships between stakeholders and align existing health programs to develop health policies. The strategies included social media and changes in community spaces and worksites as well as a school-to-family curriculum. |
| **Verbestel et al. 2015 (12)**  **De Henauw et al. 2015 (13)** | Identification and prevention of Dietary- and lifestyle-induced health EFfects In Children and infantS (IDEFICS); 8 European countries (Belgium, Cyprus, Estonia, Germany, Hungary, Italy, Spain and Sweden) | Study the effectiveness of the IDEFICS intervention measured by physical activity and sedentary time in European children | Non-randomized cluster-controlled trial using a before-after control group design. The study had two dimensions: an observational cohort (cross-sectional study) and an intervention study of a prevention program. In addition, they conducted three case-control studies with different sub-groups of children  Preschool and school-aged children (2-10 years old), and their parents  Not specified | Primary outcomes: Prevalence of overweight and obesity, anthropometric measures (BMI z-score, % body fat, WHtR), 6 behavioral outcomes and sleep duration  Secondary outcomes: PA and Sedentary Time (ST) | Socioecological approach | 2 years (2007/08-2009/10)  Family, schools/kindergarten and community spaces and facilities | PA and diet; Multi-level intervention to prevent obesity among children by increasing water consumption and fruit/vegetables, reducing TV viewing and increasing PA, as well as family time and sleep duration through activities at the level of the community, school/kindergarten and family |
| **Vinck et al. 2016 (14)** | VIASANO; French-speaking area of Belgium | Evaluate the effects of VIASANO, a community-based program using the EPODE methodology, on the prevalence of overweight in two pilot towns in Belgium | Quasi-experimental study non-randomized  Preschool children 3-4 years old and 5-6 years old, also the general public  One urban and one rural town | Anthropometric measures (weight, height, BMI, prevalence overweight/obesity) | EPODE methodology. It does not have a controlled design approach | 3 years (2007-2010)  Schools, community spaces and facilities | PA and diet; The intervention had a dual perspective: on one hand, targeting the local population via social marketing techniques using local communication channels and on the other hand, targeting the environment or community in collaboration with local actors.  For the implementation, a coordination team coaches a local project manager in each town. The project manager mobilizes local stakeholders. |
| **Ayala et al. 2015 (15) (NOT COMPLETED)** | Our Choice/Nuestra Opción, Childhood Obesity Research Demonstration study; Imperial County and USA-Mexico border, California, USA | Prevent and control obesity in a vulnerable young population living in a rural, border community. The ultimate goal is to assist children 2-12 years old to attain a healthy weight by modifying behavior within the contexts in which they occur, supported by changes to the social and structural environments | 2x2 factorial study quasi-experimental design  Children (2-11 years old) and their parents  Rural communities | Primary outcomes:  Anthropometric measures (BMI z-score), behavioral outcomes on PA and sleep.  Process, impact and outcome evaluation on children, parents and organizations | Whole-child approach, Total Worker Health, Social Ecological Framework (SEF), obesity care model, social cognitive theory, organizational change theory | 1 year (2011)  Community spaces and facilities, health care and education centers, schools | PA and diet; Multi-sector and multi-level whole-system intervention. Formative research and meetings with different representatives of the community took place to define the intervention strategies. In each community, one of the following strategies was then implemented: a) Public Health intervention; b) Health Care intervention; c) PH + HC intervention; d) Evaluation only as control. The HC intervention involved the implementation of an obesity care model in a federally qualified health center (FQHC) + family wellness program (FWP) delivered by community health workers. The PH intervention consisted in working with all the community institutions to promote healthy behaviors and prevent overweight (nutrition, water consumption, PA and quality sleep). |
| **Crespo et al. 2012 (16)** | Aventuras para Niños Study; San Diego county, California, USA | Evaluate the impact of a multi-level promotora-based (Community Health Advisor – community leaders with influence on health issues) intervention to promote healthy eating and PA and prevent excess weight gain in Latino children, but also compare the effects of changes in home/family vs school/community environments in childhood obesity | Factorial design, randomized controlled community trial  Latin children and their parents  Schools with a majority of Latino enrollment (min. 70%) | Primary outcomes:  Anthropometric measures (BMI z-score, BMI, weight)  Secondary outcomes: Behavioral outcomes (diet, PA and sports participation, sedentary behavior, parenting style for diet and activity,), demographic data (household income, household size, employment status,…) | Health belief model, social cognitive theory, structural model of health behavior | 3 years  Families, schools, community areas | PA and diet; Each of the school communities received one of the following strategies combination: a) Family-only change delivered by promotors through home visits and delivery of educational material; b) Community-only environmental change: social and physical changes in different community spaces and facilities; c) Family + Community strategies; d) No intervention (control). |
| **Pettman et al. 2014 (17)** | Eat Well Be Active (EWBA) Community Programs; South Australia | Assess the effectiveness of EWBA with respect to increasing the prevalence of healthy weight among children, and improving behaviors, attitudes and environments linked to healthy eating and physical activity, in metropolitan and regional socio-economically disadvantaged communities | Quasi-experimental before-after interventional non-randomized study and repeat cross-sectional study. Mixed-methods evaluation framework  Children (0-18 years old) and their families  Socio-economically disadvantaged communities (outer-metropolitan suburbs and rural towns) | Anthropometric measures (height, weight, BMI z-score) | Based on primary health care principles and informed by multiple health promotion theories: Socio-ecological framework with a community development and capacity building approach | 3 years (2006-2009)  Childcare and education centers, health, welfare and indigenous agencies, community spaces and facilities | PA and diet; It is a multi-setting, multi-strategy whole of community intervention to contribute to the healthy weight of children and young people and their families by working in partnership with a variety of settings to address both individual behavior and environmental barriers (infrastructure improvements, policies, social marketing and community development). Stakeholder engagement was also part of the intervention. |
| **Fotu et al. 2011 (18)** | Ma’alahi Youth Project (Pacific Obesity Prevention in Communities project – OPIC); Tonga and Vava’u, Pacific Islands | Build the capacity of communities and schools to create their own solutions for promoting healthy eating, physical activity and healthy weight gain in adolescents aged 11-19 years and their families | Quasi-experimental non-randomized study, longitudinal design  Children/adolescents (11-19 years old) and their families  Villages in different districts of the islands | Anthropometric data (height, weight, WC, BMI z-score, body fat %), prevalence of overweight/obesity, QoL | - | 3 years (2006-2008)  School, community spaces and facilities | PA and diet; The intervention was developed through the collaboration of government departments, community stakeholders and community consultation and engagement. The components of the intervention were: social marketing, activities to promote healthy nutrition and physical activity, community capacity building activities. The interventions were contextualized for each school and community. |
| **Kremer et al. 2011 (19)** | The Healthy Youth Healthy Communities (part of the OPIC project); Fiji, Pacific Islands | Strengthen the community capacity to promote healthy eating and regular PA and to reduce overweight and obesity in Fijian adolescents | Quasi-experimental non-randomized study  Children 13-18 years  Not specified | Anthropometric measures, demographic data, behavioral outcomes on eating patterns and PA, QoL, perceptions and attitudes on body size, family, home, school and neighborhood environment | - | 2/3 years (2005/06-2007/08)  Schools, community spaces and facilities | PA and diet; The intervention was implemented in schools and community settings. It aimed to increase the walking of the students, increase healthy eating, and decrease sedentary behavior. |
| **Lytvyak et al. 2016 (20)**  **Raine et al. 2013 (21)** | Healthy Alberta Communities (HAC); Alberta, Canada | Reduce lifestyle-related risk factors for chronic disease and obesity at a population-level and create environments supportive of healthier dietary and PA behaviors within four diverse communities in Alberta, Canada | Cross-sectional, non-randomized study  Free-living adults (pregnant women and individuals in wheelchairs) from 18 years old  Four communities in a geographically large, politically conservative, resource-rich province (rural towns and cities) | Anthropometric measures (height, weight, waist and hip circumference, BMI), prevalence of overweight/obesity, clinical measurements from blood samples (glucose, total cholesterol, blood pressure,…) | ANGELO framework - ecological model of health promotion to classify environments by types that influence food intake and PA | 3 years (2006-2009)  Community spaces and facilities | PA and diet; The development of the intervention took place through a community-academic partnership where four community coordinators established community networks to link the research team with each community. The intervention consisted of community gardens, promotion of leisure activities and healthy eating, changes in the environment to increase access to healthy food and community capacity building, improvement in the community networks. |
| **Zhou et al. 2014 (22)** | A policy-driven multifaceted approach for early childhood physical fitness promotion; Beijing, China | Test the effects of the intervention on healthy weight growth (body composition) and physical fitness in preschool Chinese children, and evaluate the feasibility of conducting a complex health promotion campaign in a childcare setting | Quasi-experimental before-after design with comparison group, non-randomized study  Children (3-5 years old)  Not specified | Anthropometric measures of children, physical fitness of parents, children and teachers, feasibility and fidelity of the intervention | Social-ecological models (SEM) and competence motivational theory (CMT) | 12 months (2010/11)  Public childcare centers, community spaces and facilities | PA and diet; It is a multifaceted intervention that engages childcare centers, families and the community to promote PA and support healthy eating to prevent obesity. Childcare center int.: PA policies and curriculum, teacher trainings, food services changes in the centers, training of the staff. Family int.: educational seminars on food preparation and physical activity for the family, educational materials and other family events. Community intervention: neighborhood events for PA promotion and health education, training of staff for child fitness promotion and the provision of health education in a Community Health Center. |
| **De Silva-Sanigorski et al. 2010 (23)** | Romp & Chomp; Victoria, Australia | Determine the effectiveness of the program in reducing obesity and promoting healthy eating and active play in children aged 0-5 years old as well as increase the capacity of the cities to support healthy eating and active play | Repeat cross-sectional, quasi-experimental non-randomized controlled study  Children (0-5 years old) and their families  Large regional cities | Anthropometric measures (BMI, BMI z-scores), prevalence of overweight and obesity, behavioral outcomes, environmental changes in the communities | Socio-ecological framework | 4 years (2004-2008)  Schools, care centers, maternal child health services, community health services, regional immunization services | PA and diet; Several key organizations in the region designed, planned and implemented the intervention. Several initiatives were implemented to meet the objectives, particularly professional development of workers and service staff, social marketing strategies, educational materials for parent, nutrition and PA policies in schools and kindergartens, PA demonstrations, among others. |
| **Taylor et al. 2007 (24)** | A Pilot Program for Lifestyle and Exercise (APPLE); Otago, New Zealand | Evaluate the effectiveness of a 2-y controlled community-based intervention to prevent excessive weight gain in 5–12-y-old children by enhancing opportunities for healthy eating and noncurricular physical activity | Non-randomized controlled trial (pilot study)  Children (5-12 years old)  2 semirural geographically separated communities | Anthropometric measures (height, weight, BMI z-score, WC), blood pressure, diet and physical activity behavior | NR | 2 years (2003-2005)  Schools and community | PA and diet; In the first year, community activity coordinators were assigned to each intervention school to encourage children to be physically active. Resources for teachers and the provision of cooled water filters at schools were also implemented. In the second year, science lessons to promote healthy eating and access to sport and play equipment were added to the intervention program. |
| **De Coen et al., 2012 (25)** | Prevention of Overweight among Pre-school and school children (POP); Flanders, Belgium | Evaluate the effects of a school-based, 2-year, multi-component intervention on BMI, eating and physical activity behavior in Flanders, Belgium, targeting children aged 3–6 years in communities of high and low SES | Cluster-randomized controlled trial  Pre-primary and primary school children (3-6 years old)  2 low SES, 2 medium SES and 2 high SES communities | Anthropometric measures (BMI z-scores), diet and physical activity behavior, screen time | Socio-ecological model | 2 school years (2008-2010)  Schools and community | PA and diet; At the start, two meetings were held with stakeholders for intervention planning and raising awareness. The intervention included 7 modules: Organization of the POP project at school level, organization of classroom activities, development of an active playground, implementation of health-related physical education, environmental and policy changes to increase the availability of water at school, changes to increase availability of vegetables and fruits, educational strategies for parents on all topics. Regional health boards also supported the schools. |
| **Van de Kolk et al., 2020 (26)**  **Van de Kolk et al., 2019 (27)** | SuperFIT; Limburg, the Netherlands | Increase PA levels, decrease sedentary behavior and increase healthy nutrition behavior of young children through changes in the sociocultural and physical environments | Quasi-experimental research design  Pre-school children (2-4 years old) and families  Low SES urban neighborhoods | Anthropometric measures (BMI z-score), PA and sedentary behavior | Socio-ecological models of behavior and systems theory | 1 year (2017 – 2018)  Schools, homes and community | PA and diet. SuperFIT is a comprehensive, integrated approach developed in partnership with health promotion experts and involvement of stakeholders in the development and implementation. that includes several strategies targeting the sociocultural environment and the physical environment. It consisted of a pre-school, a family-based and a community-based component. Some items included training sessions in school, delivery of nutrition and PA materials, seminars, organized family activities, among many others. The intervention was developed to be highly adaptable and integrated in the daily routines. |
| **Strugnell et al. 2024 (28)**  **Strugnell et al. 2016 (29)** | Healthy Together Victoria (HTV); Victoria, Australia | Reduce the prevalence of overweight and obesity by targeting chronic disease risk factors and chronic diseases | Cluster-randomized trial with repeated-cross-sectional evaluations  Secondary school children in grade 8 (13-15 years old) and grade 10 (15-16 years old), adults  Towns/suburbs in Victoria | Anthropometric measures (BMI z-score, WC), prevalence of overweight and obesity, PA and sedentary behavior, diet quality, HrQoL, depressive symptoms | Systems thinking (systems science) | 2 years (2014 – 2016)  Early childhood services, schools, workplaces, community, state and local level | PA and diet. The HTV was a multi-sector, multi-component whole of population approach involving a local-level workforce of 170 members. The initiative targeted multiple settings and included multiple strategies and policies at the state and local levels. It also worked with a lot of local players such as food growers, sellers, sport and recreation centers to increase access to healthy food, social marketing for health promotion. |
| **Van Dongen et al. 2022 (30)** | The Fit Lifestyle at School and at Home (FLASH); The Netherlands | Preventing overweight and building community capacity through four strategies: leadership, participatory school culture, tailored health-promotion activities and local networks. Building community capacity for creating a healthy school community. | Mixed methods, quasi-experimental design  Secondary school children  Not specified | BMI z-scores and WC, health behavior and capacity-building processes | RE-AIM framework | 3 years (2016- 2019)  Schools and community | PA and diet. FLASH focused on increasing the capacity-building in the intervention schools by identifying the needs and tailoring the strategies to each school. It included three phases: 1) assigning leadership roles, 2) mapping of needs by pupils, staff and parents, and creating context-specific solutions and action plans based on evidence-based techniques and fit within the Dutch Healthy School approach, and 3) evaluating action plans. |
| **Li et al. 2019 (31)**  **Li et al. 2017 (32)** | The Chinese Primary School Children Physical Activity and Dietary Behaviour Changes Intervention (CHIRPY DRAGON); Guangzhou, China | Preventing obesity in Chinese primary-school children | Cluster-randomized controlled trial  Children (6-7 years old) and families  Urban districts | Anthropometric measures (BMI z-score, body fat, WC), prevalence of overweight/obesity, eating and PA behaviors, psychosocial outcomes, blood pressure | United Kingdom Medical Research Council complex intervention framework | 12 months  Schools and families | PA and diet. The intervention includes a total of four components delivered by five full-time trained staff with the aim to increase the PA inside and outside of school and to improve the nutritional quality at school, as well as to improve the knowledge on obesity. The components are targeted at children, parents, teachers or school staff. Various stakeholders contributed to the choice of the intervention components. |
| **Natale et al. 2021 (33)** | Healthy Caregivers-Healthy Children (HC2); Miami, USA | Compare the effectiveness of the two phases of the HC2 trial on child dietary practices and BMI | Cluster-randomized controlled trial  Children (18 months to 5 years old) and their parents  Low-resource, ethnically diverse urban and rural communities | Dietary intake, child age and sex-adjusted BMI percentile | Social cognitive theory, RE-AIM framework | 2 years for each phase (Phase 1: 2011-2013 and Phase 2: 2015-2017)  Child-care centers (CCCs), families | PA and diet. The intervention had two phases: Phase 1 was implemented by a university-based research team; phase 2 used the train-the-trainers delivery method where teachers from the CCCs are trained to disseminate the intervention. The intervention toolkit included some diet and PA policies, lessons for children, workshops based on the principles of the “nutritional gatekeeper” concept for parents and teachers delivered by university staff. |
| **Allender et al., 2021 (34)** | WHO STOPS Childhood Obesity;  South West Victoria, Australia | Evaluate four-year  changes in BMIz, overweight and obesity  prevalence, changes in obesity-related  behaviors, and  HRQoL among children attending primary  schools in South West Victoria, Australia | Stepped-wedge cluster randomized trial (all clusters received the intervention but started at different times)  Primary school children  Not specified | Anthropometric measures (height, weight), BMIz, overweight/obesity WHO categories, diet and physical activity behavior, screen time, HRQoL (23-item  Pediatric  Quality of Life Inventory 4.0) | NR | 4 years (2015-2019)  Schools and community | PA and diet. The intervention followed a multistage, community-driven process to address childhood obesity, beginning with data collection and stakeholder engagement, followed by the creation of a systems map to identify key drivers. Community leaders and organizations then collaboratively designed and implemented targeted actions, such as healthier food policies and infrastructure improvements, while ongoing data collection supported adaptation and sustainability. |
| **Buch-Andersen et al., 2021 (35)** | The Danish SoL Project; Bornholm (intervention) and Odsherred (control), Denmark | Evaluate the effect of Project SoL on anthropometric  measures related to overweight in three- to eight-year-old children in two municipalities in Denmark over 19 months | Quasi-experimental study  Children (3-8 years old)  Low SES communities with a high prevalence of health risk factors | Anthropometric measures (height, weight), BMIz, underweight, healthy weight, overweight, obesity classified | Supersetting approach and action research methodology | 19 months (September 2012 – April 2014)  Childcare centers, schools, supermarkets and local communities | PA and diet. Project SoL was a four-year initiative with a 19-month intervention period to promote physical activity and healthy eating among young children and their families. The intervention took place in childcare centers, schools, supermarkets, media, and local communities, using educational activities, community events, social media, and environmental strategies. It was developed together with citizens and professionals, avoiding a focus on child weight to prevent stigma. |
| **French et al., 2023 (36)** | NET-Works; Minneapolis-Saint Paul, Minnesota, USA | Effect of a multi-setting, multi-component, community-based pediatric obesity prevention intervention on child BMI and other cardiometabolic risk factors | Randomized controlled trial  Children (2-4 years old at time of inclusion) and their parents  Low-income, racial/ethnic minority families | Anthropometric measures (height, weight), BMI, blood pressure, metabolic parameters, dietary intake, physical activity, screen time | Social ecological theory | 66 months (2012–2018)  Primary care clinics, homes, neighborhood | PA and diet. The intervention, based on social ecological theory and prior research, included home visits, community-based parenting classes, and telephone check-ins. Trained professionals delivered the program using Motivational Interviewing, focusing on parents as agents of change. Strategies included goal setting, behavioral tracking, and modifying home environments to promote healthy routines. Intervention dose was consistent across three years, with referrals to community resources integrated throughout. |
| **Gago et al., 2023 (37)** | Communities  for Healthy Living (CHL) using Head Start programs (Greater Boston Head Start); Cambridge and Somerville, MA, USA | Effect of CHL on BMIz and weight-related behaviors (i.e., diet, physical activity, sleep, media use) | Stepped-wedge cluster randomized trial (all clusters received the intervention but started at different times)  Children (33 months to 5 years old) and their families  Low-income families | Anthropometric measures (height, weight), BMI z-score, child health behaviors, parental empowerment and parenting  practices | Community-based Participatory Research (CBPR), Family Ecological Model, empowerment theory | 3 years (2017-2019)  2 years of data collection due to COVID-19 pandemic  (media campaign and enhanced nutrition support only for six months out of 10, and no programs implemented PConnect in year 3, so only data until year 2 was decided to be evaluated because the overall intervention exposure was only the half)  Families and Head Start Centers | PA and diet. The CHL intervention enhanced standard Head Start practices (a federally-funded school readiness program for young children) by providing additional nutrition support, a media campaign, and an empowerment-focused parenting program. It targeted children's diet, physical activity, sleep, media use, and healthy body weight. Each component built on existing Head Start infrastructure with novel CHL innovations. |

*EPODE: Successful intervention developed in 2004 in France as a CB program that included environmental strategies to stimulate long-term, sustainable behavioral change and prevent childhood obesity. Abbreviations: *aOR – adjusted Odds Ratio, BMI – body mass index, CB – community based, CDC – Center for Disease Control, CI – confidence interval, EPODE – Ensemble Prévenons l’Obésité des Enfants, HRQoL – Health-related quality of life, IOTF – International Obesity Task Force, NICE, PA – physical activity, PESTEL, RE-AIM – Reach Effectiveness Adoption Implementation Maintenance, SD – standard deviation, SE – standard error, SES – socioeconomic status, WHO – World Health Organization, WtHR – waist to hip ratio, ys – years*

Table S4. Overview of results of the included studies.

| **Study, Authors, Year** | **Data collection time points** | **N of control communities and participants** | **N of intervention communities and participants** | **Intervention (absolute difference baseline vs. follow-up)*** | **Control (absolute difference baseline vs. follow-up)*** | **Effect size: Intervention effect (intervention vs. control)** | **Overall effects summary (intervention compared to control)** |
| --- | --- | --- | --- | --- | --- | --- | --- |
| **Shape Up Somerville**  **Coffield et al. 2015 (1)**  **Economos et al. 2007 (2)**  **Economos et al. 2013 (3)** | Baseline, 1 year and 2-years follow-up | Baseline:  2 communities: 20 schools (15 in Control 1, 5 in Control 2): n = 890 children analyzed, n = 1,003 parents of 567 households  analyzed  Follow-up:  n = 793 children analyzed (n = 561 in Control 1, n = 232 in Control 2) at year 1, and n = 693 at year 2,  n = 507 parents analyzed at year 2 | Baseline:  1 community: 10 schools: n = 461 children analyzed, n = 527 parents of 293 households  Follow-up:  n = 385 children analyzed at year 1, n = 335 children at year 2, n = 162 parents analyzed at year 2 | 1) BMI z-score (CDC, unadjusted)  Baseline:  Males: 0.918 SD 1.021; females: 0.782 SD 1.100  Follow-up (reported for 1 year only):  Males: 0.882 SD 1.022; females: 0.755 SD 1.070  Difference:  Males: -0.036 SD 0.284; females: -0.027 SD 0.356  2) Prevalence overweight/obesity (%)  Baseline:  Males: 46.8%; females: 43.3%  Follow-up (at 2 years):  Males: 50.3%, females: 42.7%  Difference:  Males: 3.5%; females: -0.6% | 1) Baseline (Comm. 1 and 2 respectively):  Males: 0.777 SD 0.999 and 1.132 SD 0.903; females: 0.617 SD 1.060 and 0.679 SD 1.055  Follow-up (reported for 1 year only):  Males: 0.768 SD 0.995 and 1.113 SD 0.926; females: 0.615 SD 1.065 and 0.688 SD 1.055  Difference:  Males: -0.009 SD 0.289 and -0.018 SD 0.253; females: -0.002 SD 0.265 and 0.009 SD 0.294  2) Baseline:  Males: 39.9%; females: 35.4%  Follow-up (at 2 years):  Males: 46.9%; females: 39%  Difference:  Males: 7%; females: 3.6% | 1) BMI z-score after adjusting for the covariates and pooling the two control communities:  -0.1005 (95% CI  -0.1541, -0.0555; p = 0.001) at 1 year, -0.06 (95% CI -0.08, -0.04; p = 0.005) at 2 years  Compared to control 1: -0.1307 (95% CI -0.1836,  -0.0778; p = 0.02) at 1 year, -0.05 (95% CI -0.13, 0.02; p = 0.07) at 2 years  Compared to control 2: -0.1005 (95% CI -0.1151,  -0.0859; p = 0.001) at 1 year, -0.06 (95% CI -0.1, 0.03; p = 0.03) at 2 years  2) aOR:  Overall: 0.71 (95% CI 0.56, 0.90; p = 0.004); males: 0.61 (p = 0.01); females: 0.78 (p = 0.01) at 2 years  Covariates included sex, age, grade, primary language, race/ethnicity, child BMIz at baseline and clustering on community. | Statistically significant intervention effects in the reduction of BMI z-scores and overweight/obesity prevalence after 1 and 2 years. |
| **OPAL**  **Bell et al. 2019 (4)** | Baseline and 5 years follow-up  Evaluation period was shortened due to initial delays to 2-3 years intervention | Baseline:  12 lower SES (Index of Relative Socio-Economic Disadvantage-IRSD) communities (phase 1) and 8 communities (phase 2): n = 1,238 completed surveys, n = 1,145 for measurements  Follow-up:  6 communities (phase 1) and 4 communities (phase 2): n = 781 completed surveys, n = 750 for measurements | Baseline:  6 communities (phase 1) and 4 communities (phase 2): n = 1,373 completed surveys, n = 1,208 for measurements  Follow-up:  6 communities (phase 1) and 4 communities (phase 2): n = 1,092 completed surveys, n = 1,010 for measurements | 1) BMI z-score (measured by trained data collectors, mean of two measures, UK 1990 reference data)  Baseline:  0.33  Follow-up:  0.40  Difference:  0.07  2) Prevalence of overweight (%)  Baseline:  18.1%  Follow-up:  18.7%  Difference:  0.6%  3) Prevalence of obesity (%)  Baseline:  5.5%  Follow-up:  4.6%  Difference:  -0.9% | 1) Baseline:  0.30  Follow-up:  0.46  Difference:  0.16  2) Baseline:  15.8%  Follow-up:  17.9%  Difference:  2.1%  3) Baseline:  3.9%  Follow-up:  6.5%  Difference:  2.6% | 1) -0.08 (95% CI -0.24, 0.08; p = 0.31)  (marginal mean difference adjusted for SES)  2) aOR 0.87 (95% CI 0.6, 1.25; p > 0.05) (adjusted for SES and age)  3) aOR 0.51 (95% CI 0.28, 0.92; p > 0.05) (adjusted for SES and age)  After Bonferroni adjustment. | No statistically significant differences after Bonferroni adjustment in changes in the likelihood of obesity, BMI z-score, but the intervention group was favored in these outcomes. |
| **POIBC-TCHP**  **Gomez et al. 2018 (5)** | Baseline and 15 months follow-up | Baseline:  2 towns (n = 1,112)  Follow-up:  2 towns (n = 1,112) | Baseline:  2 towns (n = 1,041)  Follow-up:  2 towns (n = 974) | 1) BMI sex-specific z-score  Baseline:  0.73 SD 1.20  Follow-up:  0.64 SD 1.12  Difference:  -0.09 | 1) Baseline  0.67 SD 1.15  Follow-up:  0.57 SD 1.15  Difference:  -0.1 | 1) 0.078; p = 0.94 (adjusted by gender, age, lifestyle variables at baseline) and 0.012; p = 0.73 (adjusted by gender, age, maternal education and baseline values of other indexes) | No statistically significant intervention effects in BMIz, but reductions in these outcome were observed in both groups. |
| **Be Active Eat Well (BAEW)**  **Swinburn et al. 2014 (7)**  **Johnson et al. 2012 (8)**  **Sanigorski et al. 2008 (9)** | Baseline and 3 years post-intervention | Baseline:  1 community of 320,000 inhabitants: 10 primary schools (n = 797)  Follow-up:  n = 621 | Baseline:  1 town of 11,000 inhabitants: 6 primary schools (n = 877)  Follow-up:  n = 660 | 1) BMI  Baseline:  18.0 SD 2.8  Follow-up:  17.8 SD 2.9  Difference:  -0.2  2) BMI z-score (WHO criteria, adjusted for gender and age)  Baseline:  0.79 SD 1.07  Follow-up:  0.65 SD 1.10  Difference:  -0.14  3) Prevalence overweight/obesity (WHO criteria)  Baseline:  39.2%  Follow-up:  32.8%  Difference:  -6.35 (95% CI -11.15, -1.46) | 1) Baseline:  18.0 SD 2.7  Follow-up:  17.5 SD 2.7  Difference:  -0.5  2) Baseline:  0.77 SD 1.04  Follow-up:  0.57 SD 1.0  Difference:  -0.2  3) Baseline:  39.6%  Follow-up:  29.1%  Difference:  -10.43 (95% CI -15.33, -5.42) | NR (no values were reported) | No statistically significant differences between groups in BMI, BMIz and prevalence of overweight/ obesity, but larger reductions were observed in the control area.  If control and intervention are pooled, there is a significant decrease in the BMIz and overweight/obesity prevalence (p < 0.001) from baseline to follow-up. |
| **HCP: BAEW**  **Bolton et al. 2017 (10)** | Baseline and follow-up (12 months to 2 years) | Baseline:  18 primary schools, 15 secondary schools, 7 workplaces  Follow-up:  17 primary schools (n = 1028), 14 secondary schools (n = 351), 7 workplaces | Baseline:  5 communities: 18 primary schools, 15 secondary schools, 7 workplaces  Follow-up:  5 communities: 17 primary schools (n = 904), 14 secondary schools (n = 681), 7 workplaces (n = 135) | 1) BMI z-score (WHO guidelines)  Primary schools: 0.58 SE 0.07;  Secondary schools: 0.35 SE 0.09; Workplaces: NR  2) Prevalence overweight/obesity  Follow-up:  Primary schools (n = 1028): 36.7%; Secondary schools (n = 351): 25.8%; Workplaces (n = 155): 54.1% | 1) Primary schools: 0.63 SE 0.06; Secondary schools: 0.34 SE 0.03; Workplaces: NR  2) Primary schools (n = 904): 34.8%; Secondary schools (n = 681): 27.3%; Workplaces (n = 133): 50.0% | 1) -0.03 (Comm. 1),  -0.03 (Comm. 2), 0.05 (Comm. 3), -0.29 (Comm. 4), NR (Comm. 5)  Primary schools (Comm. 1 and 2):  -0.02  Secondary schools: 0.01  Workplaces: NR  All p values > 0.05 except for Comm. 4.  Adjusted for age, gender, level of disadvantage, baseline means and clustering by school or workplace.  2) NR | Statistically significant differences for BMI z-score only for Community 4 (p < 0.05) in favor of the intervention, but not for the rest of communities or when all communities were pooled together. |
| **IDEFICS**  **Verbestel et al. 2015 (12)**  **De Henauw et al. 2015 (13)** | Baseline and 2 years follow-up | Baseline:  1 area per country: 8 to 35 schools per country (n = 7,746)  Follow-up:  n = 5,314 | Baseline:  1 area per country: 5 to 42 schools per country (n= 8,842)  Follow-up:  n = 5,727 | 1) BMI z-scores (IOTF references)  Baseline:  Boys: 0.439 SE 0.138; girls: 0.300 SE 0.137  Follow-up:  Boys: 0.553 SE 0.138; girls: 0.401 SE 0.137  Difference:  Boys: 0.3837; girls: 0.162  2) Prevalence overweight/obesity (%)  Baseline:  19%  Follow-up:  23.6%  Difference:  4.6% | 1) Baseline:  Boys: 0.377 SE 0.138; girls: 0.251 SE 0.137  Follow-up:  Boys: 0.532 SE 0.138; girls: 0.447 SE 0.137  Difference:  Boys: 0.155; girls: 0.196  2) Baseline:  18%  Follow-up:  22.9%  Difference:  4.9% | No values were reported overall, only stratified by gender:  1) Boys: -0.043 (p = 0.333)  Girls: -0.095 (p = 0.042)  2) NR (values were not reported)  Adjusted for age and SES of the parents, corrected for cluster design | No statistically significant differences between groups for prevalence of overweight/obesitywith all schools pooled.  Statistically significant lower increase of BMI z-score among girls in the intervention group compared to the control group. |
| **VIASANO**  **Vinck et al. 2016 (14)** | Baseline and 3 years follow-up | The rest of the French speaking area in Belgium n = 76,864 (baseline) and  n = 79,692 (follow-up) | Baseline  2 towns  Follow-up:  n = 1,484 | 1) Prevalence of overweight/obesity (%); overweight = above the 85th percentile, obesity = above the 97^th^ percentile (Flemish growth curves), adjusted for sex and school grade  Baseline:  13.6%  Follow-up:  11.2%  Difference:  -2.2% (95% CI -4.6%, -0.3%; p = 0.082) | 1) Baseline:  15.8%  Follow-up:  16.0%  Difference:  0.2% | NR (only the p value was reported: p = 0.058) | No statistically significant differences between groups, but results were favorable for the intervention group (decrease in the prevalence of overweight/obesity observed). |
| **Aventuras para Niños Study**  **Crespo et al. 2012 (16)** | Baseline (M1), 1 year post-intervention (M2), 1 year (M3) and 2 years (M4) follow-up | Baseline:  4 schools: M1 (n = 223)  Follow-up:  M2: n = 205; M3: n = 171; M4: n = 134 | Baseline:  3 schools: M1 (n = 195)  Follow-up:  3 schools:  M2: n = 167;  M3: n = 140;  M4: n = 96 | 1) BMI z-score (CDC 2000 growth charts)  M1: 0.94 SD 1.23 (Fam-only); 0.87 SD 1.11 (Comm-only); 0.86 SD 1.12 (Fam+Comm); M2: 0.95 SD 1.13, M3: 1.01 SD 1.08, M4: 1.00 SD 1.04 (Fam-only); M2: 0.85 SD 1.09, M3: 0.93 SD 1.01, M4: 0.99 SD 1.05 (Comm-only); M2: 0.86 SD 1.06, M3: 0.94 SD 0.99, M4: 0.95 SD 1.05 (Fam+Comm)  Difference-(M4-M1):  +0.6 (Fam-only); +1.2 (Comm-only); +0.9 (Comm+Fam)  2) Overweight prevalence (%, 85^th^ to 95^th^ percentile weight for age)  M1: 14% (Fam-only); 19% (Comm-only); 19% (Fam+Comm); M2: 17%, M3: 22%, M4: 23% (Fam-only); M2: 19%, M3: 21%, M4: 20% (Comm-only); M2: 17%, M3: 21%, M4: 18% (Fam+Comm)  Difference (M4-M1):  +9% (Fam-only); +1% (Comm-only); -1% (Fam+Comm)  3) Obesity prevalence (%, 95^th^ percentile weight for age)  M1: 31% (Fam-only); 28% (Comm-only); 27% (Fam+Comm); M2: 29%, M3: 29%, M4: 30% (Fam-only); M2: 26%, M3: 27%, M4: 35% (Comm-only); M2: 27%, M3: 26%, M4: 32% (Fam+Comm)  Difference (M4-M1):  -1% (Fam-only); +7% (Comm-only); +5% (Fam+Comm) | 1) M1: 1.00 SD 1.10; M2: 0.99 SD 1.12;  M3: 1.03 SD 1.08; M4: 0.97 SD 1.09  Difference (M4-M1):  -0.3  2) M1: 18%; M2: 17%; M3: 17%; M4: 13%  Difference (M4-M1):  -5%  3) M1: 31%, M2: 33%; M3: 35%; M4: 35%  Difference (M4-M1):  +4% | NR (no values were reported) | No statistically significant changes in any of the weight measures. Greatest increase of overweight prevalence observed for Fam-only and Comm-only groups. Prevalence of obesity increased in all groups except in Fam-only. There were also no significant intervention effects on parents BMI or BMI category. Intervention effects did not vary after adding interaction terms (baseline weight status and child gender). All children increased BMI z-score over the course of the study in all groups. |
| **EWBA**  **Pettman et al. 2014 (17)** | Baseline and 3 years follow-up | Baseline:  2 communities (1 metropo-litan, 1 rural) (n = 541)  Follow-up:  2 communities (n = 789) | Baseline:  2 communities (1 metropolitan, 1 rural) (n = 1,300)  Follow-up:  2 communities (n = 1,005) | 1) BMI z-score (reference data by Cole et al. 1995 and McCarthy et al. 2001)  Baseline:  0.64 SD 1.07  (4-5 year-olds);  0.596 SD 1.127  (10-12 year-olds)  Follow-up:  0.44 SD 1.07  (4-5 year-olds);  0.595 SD 1.108  (10-12 year-olds)  Difference:  -0.20 (4-5 year-olds); -0.02 (10-12 year-olds)  2) Prevalence of overweight (%, BMI IOTF references)  Baseline:  19.4% (95% CI 16.1, 23.2)  (4-5 year-olds); 16.7% (95% CI 14.5, 19.6) (10-12 year-olds)  Follow-up:  17.8% (95% CI 14.6, 21.6)  (4-5 year-olds); 21.8% (95% CI 18.7, 25.4) (10-12 year-olds)  Difference:  -1.6% (4-5 year-olds); 5.0% (10-12 year-olds)  3) Prevalence of obesity (%, BMI IOTF references)  Baseline:  8.8% (95% CI 6.6, 11.8)  (4-5 year-olds); 7.5% (95% CI 5.8, 9.4) (10-12 year-olds)  Follow-up:  4.2% (95% CI 2.7, 6.4); 5.5% (95% CI 4, 7.7) (10-12 year-olds)  (4-5 year-olds);  Difference:  -4.7% (4-5 year-olds); -1.9% (10-12 year-olds) | 1) Baseline:  0.50 SD 1.02  (4-5 year-olds); 0.598 SD 1.115  (10-12 year-olds)  Follow-up:  0.35 SD 1.10  (4-5 year-olds); 0.548 SD 1.131  (10-12 year-olds)  Difference:  -0.15 (4-5 year-olds); -0.050 (10-12 year-olds)  2) Baseline:  17.6% (95% CI 14.6, 21.0)  (4-5 year-olds); 20.1% (95% CI 17.5, 23.1) (10-12 year-olds)  Follow-up:  14.3% (95% CI 12.1, 16.9)  (4-5 year-olds); 19.9% (95% CI 16.9, 23.3) (10-12 year-olds)  Difference:  -3.2% (4-5 year-olds); -0.2 (10-12 year-olds)  3) Baseline:  5.4% (95% CI 3.8, 7.6)  (4-5 year-olds); 5.7% (95% CI 4.3, 7.5) (10-12 year-olds)  Follow-up:  4.8% (95% CI 3.5, 6.5); 5.2% (95% CI 3.7, 7.3) (10-12 year-olds)  Difference:  -0.5% (4-5 year-olds); -0.5% (10-12 year-olds) | NR (no values were reported) | Statistically significant decrease in BMIz in both intervention and control groups for 4-5 year-olds, but these changes were not statistically significantly different between groups (p < 0.05). Larger decrease (statistically significant, p < 0.05) in overweight/obesity prevalence in the intervention compared to control among 4-5 year-olds.  No statistically significant changes in the outcomes for 10-12 year-olds, but larger decrease in obesity prevalence in the intervention than in the comparator group. |
| **Ma’alahi Youth Project**  **Fotu et al. 2011 (18)** | Baseline and 3 years follow-up | Baseline:  6 schools (n = 1,396)  Follow-up:  6 schools (n = 897) | Baseline:  3 districts with 22 villages and 7 schools (n = 1,083)  Follow-up:  n = 815 | 1) BMI z-scores (WHO 2007 standards for age/gender specific BMI centiles and cut-offs)  Baseline  0.9 SD 0.9  Follow-up:  1.1 SD 0.9  Difference:  0.2  2) Prevalence overweight (%)  Baseline:  30.8%  Follow-up:  36.6%  Difference:  5.8%  3) Prevalence of obesity (%)  Baseline:  15.6%  Follow-up:  19.9%  Difference:  4.3% | 1) Baseline:  0.9 SD 1.0  Follow-up:  1.1 SD 0.9  Difference:  0.2  2) Baseline:  32.2%  Follow-up:  39.9%;  Difference:  7.6%  3) Baseline:  14.0%  Follow-up:  18.8%  Difference:  4.8% | 1) Adjusted:  -0.03 SE 0.03; p = 0.26  2) Adjusted pooled overweight/obesity (OR): -0.05 SE 0.24; p = 0.84  Adjusted for baseline variable, age at follow-up, height at follow-up, gender and time between measurements | No statistically significant intervention effects in these outcomes. |
| **The Healthy Youth Healthy Communities**  **Kremer et al. 2011 (19)** | Baseline and 2-years follow-up | Baseline: 3 towns (11 secondary schools) n = 4,567  Follow-up: 3 towns (11 secondary schools) n = 2,069 | Baseline: 1 community (7 schools) n = 2,670  Follow-up: 1 community (7 schools) n = 879 | 1) BMI z-score (WHO 2007 reference)  Baseline:  -0.30 SD 1.42  Follow-up:  -0.34 SD 1.45  Difference:  -0.04  2) Prevalence overweight (%) (WHO cut-off points)  Baseline:  12.7%  Follow-up:  12.8%  Difference:  0.1  3) Prevalence obesity (%) (WHO cut-off points)  Baseline:  5.5%  Follow-up:  5.3%  Difference:  -0.2 | 1) Baseline:  -0.01 SD 1.35  Follow-up:  -0.03 SD 1.35  Difference:  -0.02  2) Baseline:  16.5%  Follow-up:  15.3%  Difference:  -1.2%  3) Baseline:  6.2%  Follow-up:  6.0%  Difference:  -0.2% | 1) 0.02 SE 0.02  (95% CI -0.02, 0.07; p = 0.33)  2) Proportion overweight/obesity pooled: 0.34 SE 0.19  95% CI -0.03, 0.71; p = 0.07  Adjusted for baseline measure, gender, ethnic subgroup, age and height at follow-up, duration between measures and clustering by school. | No statistically significant differences in these outcomes between groups. |
| **Healthy Alberta Communities**  **Lytvyak et al. 2016 (20)**  **Raine et al. 2013 (21)** | Baseline and 3 years follow-up | Baseline:  National sample survey n = 3,725  Follow-up:  n = 3,873 | Baseline: 4 communities (n = 1,554)  Follow-up:  4 communities (n = 1,808) | 1) BMI  Baseline:  20-39 years: 26.69 (95% CI 26.17-27.21); 40-59 years: 28.79 (95% CI 28.32-29.26); 60-79 years: 29.24 (95% CI 28.60-29.88)  Follow-up:  20-39 years: 26.29 (95% CI 26.23-27.13); 40-59 years: 28.79 (95% CI 28.32-29.26); 60-79 years: 29.62 (95% CI 29.00-30.23)  Difference:  20-39 years: -0.4; 40-59 years: 0.00; 60-79 years: 0.38  2) Prevalence overweight (BMI 25.0-29.9)  Baseline:  18-79 years: 35.3 (95% CI 32.8-37.7)  Follow-up:  18-79 years: 34.5 (95% CI 32.2-36.8)  Difference:  -0.8%  3) Prevalence obesity class I (BMI 30.0-34.9), class II (BMI 35.0-39.9), class III (BMI>40)  Baseline:  Class I: 18-79 years: 18.0 (95% CI 16.0-19.9)  Class II: 18-79 years: 7.4 (95% CI 6.1-8.8) Class III: 18-79 years: 4.0 (95% CI 3.0-5.1)  Difference:  Class I: 1.3%; class II: 0.2%; class III: 0.1% | 1) Baseline:  20-39 years: 26.20 (95% CI 25.64-26.77); 40-59 years: 27.69 (95% CI 27.18-28.19); 60-79 years: 28.23 (95% CI 27.85-28.62)  Follow-up:  20-39 years: 25.87 (95% CI 25.09-26.65); 40-59 years: 28.02 (95% CI 27.38-28.66); 60-79 years: 28.43 (95% CI 27.75-29.12)  Difference:  20-39 years: -0.33; 40-59 years: 0.33; 60-79 years: 0.2  2) Baseline:  18-79 years: 36.7 (95% CI 33.7-39.7)  Follow-up:  18-79 years: 34.2 (95% CI 30.9-37.7)  Difference:  -2.5%  3) Baseline:  Class I: 18-79 years: 15.1 (95% CI 13.3-16.9)  Class II: 18-79 years: 5.8 (95% CI 4.7-7.0) Class III: 18-79 years: 3.0 (95% CI 2.3-3.7)  Difference:  Class I: 1.1%; class II: 0.5%; class III: 0.7% | 1) NR (only the p value was reported: p > 0.05)  2) NR (only the p value was reported: p > 0.05)  3) NR (only the p value was reported: p > 0.05) | No statistically significant differences between groups in these outcomes. |
| **A policy-driven multifaceted approach for early childhood physical fitness promotion**  **Zhou et al. 2014 (22)** | Baseline and 12 months follow-up | Baseline:  1 childcare center (n = 225)  Follow-up:  1 childcare center (n = 218) | Baseline:  1 childcare center (n = 148)  Follow-up:  1 childcare center (n = 139) | 1) BMI  Baseline:  15.76 SE 0.09  Follow-up:  16.04 SE 0.10  Difference:  0.28  2) BMI for age z-score (IOTF reference scores)  Baseline:  0.26 SE 0.07  Follow-up:  0.44 SE 0.07  Difference:  0.18 | 1) Baseline:  15.64 SE 0.12  Follow-up:  15.78 SE 0.14  Difference:  0.14  2) Baseline:  0.19 SE 0.08  Follow-up:  0.26 SE 0.09  Difference:  0.07 | 1) 0.19 (95% CI -0.06, 0.43; p > 0.05)  2) 0.15 (95% CI -0.01, 0.31; p > 0.05)  Adjusted for baseline measure | No statistically significant differences between groups in these outcomes, but larger increase of BMI and BMI z-score in the intervention group. |
| **Romp & Chomp**  **De Silva-Sanigorski et al. 2010 (23)** | Baseline and 3 years follow-up | Baseline:  59 communities (n = 17,732 for 2 year-olds and n = 14,647 for 3.5 year olds)  Follow-up:  59 communities (n = 21,911 for 2 year-olds and 19,050 for 3.5 year-olds) | Baseline  2 communities (n = 1,587 for 2 year-olds and n = 1,194 for 3.5 year-olds)  Follow-up:  2 communities (n = 1,611 for 2 year-olds and n = 1,239 for 3.5 year-olds) | 1) BMI  Baseline:  16.84 SE 0.04 (2 ys); 16.35 SD 0.05 (3.5 ys)  Follow-up:  16.77 SE 0.04 (2 ys); 16.17 SD 0.04 (3.5 ys)  Difference:  -0.07 (2 ys); -0.18 (3.5 ys)  2) BMI z-score (CDC 2000 growth charts and IOFT weight status)  Baseline:  0.71 SE 0.03 (2 ys); 0.67 SE 0.03 (3.5 ys)  Follow-up:  0.68 SE 0.03 (2 ys); 0.54 SE 0.03 (3.5 ys)  Difference:  -0.03 (2 ys); -1.3 (3.5 ys)  3) Overweight prevalence (%)  Baseline:  13.8 SE 0.9 (2 ys); 14.4 SE 1.1 (3.5 ys)  Follow-up:  12.5 SE 0.9 (2 ys); 12.7 SE 1.0 (3.5 ys)  Difference:  -1.3 (2 ys); -1.7 (3.5 ys)  4) Obesity prevalence (%)  Baseline:  3.3 SE 0.5 (2 ys); 4.3 SE 0.6 (3.5 ys)  Follow-up:  2.1 SE 0.4 (2 ys); 2.6 SE 0.4 (3.5 ys)  Difference:  -1.2 (2 ys); -1,.7 (3.5 ys) | 1) Baseline:  16.60 SD 0.01 (2 ys); 16.20 SD 0.01 (3.5 ys)  Follow-up:  16.57 SD 0.01 (2 ys); 16.17 SD 0.01 (3.5 ys)  Difference:  -0.03 (2 ys); -0.03 (3.5 ys)  2) Baseline:  0.54 SE 0.01 (2 ys); 0.56 SE 0.01 (3.5 ys)  Follow-up:  0.52 SE 0.01 (2 ys); 0.54 SE 0.01 (3.5 ys)  Difference:  -0.02 (2 ys); -0.02 (3.5 ys)  3) Baseline:  11.2 SE 0.3 (2 ys); 13.2 SE 0.3 (3.5 ys)  Follow-up:  10.8 SE 0.2 (2 ys); 12.7 SE 0.3 (3.5 ys)  Difference:  -0.4 (2 ys); -0.5 (3.5 ys)  4) Baseline:  2.0 SE 0.1 (2 ys); 3.2 SE 0.2 (3.5 ys)  Follow-up:  1.7 SE 0.1 (2 ys); 3.0 SE 0.1 (3.5 ys)  Difference:  -0.3 (2 ys);  -0.2 (3.5 ys) | NR (no values were reported) | Statistically significant reductions (p < 0.05) in weight status, BMI and BMIz from baseline to follow-up in the intervention group.  Statistically significant shift in the distribution of weight status in the intervention sample, where a higher proportion of children were in the healthy-weight range in both age groups. |
| **APPLE Project**  **Taylor et al. 2007 (24)** | Baseline, 1-year and 2-years follow-up | Baseline: 3 schools (n = 219 children analyzed)  Follow-up: 3 schools (n = 217 analyzed) at year 1 and 3 schools (n = 137 children analyzed) at year 2 | Baseline: 4 schools (n = 251 children analyzed)  Follow-up: 4 schools (n = 247 children analyzed) at year 1 and 4 schools (n = 151 children analyzed) at year 2 | 1) BMI  Baseline:  17.4 SD 2.4  Follow-up:  17.9 SD 2.7 (year 1); 18.3 SD 3.1 (year 2)  Difference:  0.9 (year 2)  2) BMI z-score (CDC 2000 references)  Baseline:  0.61 SD 0.82  Follow-up:  0.53 SD 0.84 (year 1); 0.45 SD 1.00 (year 2)  Difference:  -0.08 (year 1); -0.16 (year 2)  3) Overweight prevalence (%) (BMI ≥ 85^th^ percentile)  Baseline:  32.4%  Follow-up:  28.0% (year 1); 28.5% (year 2)  Difference:  -3.9% (year 2) | 1) Baseline:  18.2 SD 3.3  Follow-up:  18.9 SD 3.8 (year 1); 17.9 SD 2.7 (year 2)  Difference:  -0.3 (year 2)  2) Baseline:  0.8 SD 0.87  Follow-up:  0.79 SD 0.86 (year 1); 0.89 SD 0.81 (year 2)  Difference:  -0.01 (year 1); 0.09 (year 2)  3) Baseline:  42.5%  Follow-up:  40.6% (year 1); 47.8% (year 2)  Difference:  -5.3% (year 2) | 1) NR (no values were reported)  2) Mean difference:  -0.09 (95% CI -0.18,  -0.01) at year 1; -0.26 (95% CI -0.32, -0.21) at year 2 (p values not reported)  3) RR int. vs. control: 0.92 (95% CI 0.71, 1.18) at year 1; 0.88 (95% CI 0.69, 1.14) at year 2 (p values not reported) | Statistically significant differences between groups in BMI z-scores when adjusted for baseline values, clustering, age, sex, activity rating and television viewing. Statistically significant interaction between intervention group and classification of overweight (when stratified by weight, intervention effect was only observed in overweight children).  No statistically significant differences for overweight prevalence once adjusted for baseline values. |
| **Prevention of overweight (POP)**  **De Coen et al., 2012 (25)** | Baseline and 2-years follow-up | Baseline:  3 communities (13 schools, n = 2416 children)  1 community low SES, 1 community med SES and 1 community high SES  Follow-up:  3 communities (13 schools, n = 298 questionnaires analyzed, n = 442 height/weight measured) | Baseline:  3 communities (18 schools, n = 2034 children)  1 community low SES, 1 community med SES and 1 community high SES  Follow-up:  3 communities (18 schools, n = 396 questionnaires analyzed, n = 670 weight/height measured) | 1) BMI (Flemmish reference data)  Baseline:  15.9 SD 1.52  Follow-up:  15.7 SD 1.46 (high SES 15.9 SD 1.65, med SES 15.9 SD 1.42, low SES 15.9 SD 1.42)  Difference:  -0.2  2) BMI z-score  Baseline:  0.12 SD 0.95 (high SES 0.09 SD 1.03, med SES 0.17 SD 0.88, low SES 0.11 SD 0.92)  Follow-up:  0.11 SD 1.03 (high SES 0.17 SD 0.95, med SES 0.23 SD 0.95; low SES 0.00 SD 1.11)  Difference:  -0.01 | 1) Baseline:  15.9 SD 1.52  Follow-up:  15.9 SD 1.37  (high SES 15.9 SD 1.35, med SES 15.8 SD 1.31, low SES 15.9 SD 1.38)  Difference:  0  2) Baseline:  0.13 SD 0.92 (high SES 0.18 SD 0.86, med SES 0.09 SD 0.89, low SES 0.12 SD 0.95)  Follow-up:  0.15 SD 0.89 (high SES 0.15 SD 0.97, med SES 0.11 SD 0.83, low SES 0.16 SD 0.86)  Difference:  0.02 | 1) NR (no values were reported)  2) Only reported for the low SES group:  -0.46 statistical effect size with a statistical power of 0.80, p < 0.01 | Intervention effect not statistically significant for the total sample, only for the low SES communities (p<0.01). |
| **SuperFIT**  **Van de Kolk et al., 2019 (27)**  **Van de Kolk et al., 2020 (26)** | Baseline, 3-months and 1-year follow-ups | Baseline: 1 community (9 schools, n = 92 children)  Follow-up:  Not specified | Baseline: 1 community (12 schools, n = 47 for full intervention including the family component and n = 52 for partial intervention excluding the family component)  Follow-up:  Not specified | 1) BMI z-score (Dutch reference population)  Baseline:  Full intervention 0.25 SD 1.02; partial intervention 0.16 SD 0.88  Follow-up:  Full intervention 0.20 SD 0.98; partial intervention 0.20 SD 0.81 (3 months)  Full intervention 0.28 SD 0.90; partial intervention -0.01 SD 0.77 (1 year)  Difference:  Full intervention -0.05; partial intervention 0.04 (3 months)  Full intervention 0.03; partial intervention  -0.17 (1 year)  Measurements were not available for all children participating | 1) Baseline:  0.13 SD 0.96  Follow-up:  0.13 SD 1 (3 months); 0.08 SD 0.94 (1 year)  Difference:  0 (3 months); -0.05 (1 year)  Measurements were not available for all children participating | 1) Control vs. full intervention:  -0.09 (95% CI -0.31, 0.13; p = 0.44), ES = -0.09 (3 months)  0.00 (95% CI -0.25, 0.25; p = 0.99), ES = 0.01 (1 year)  Control vs. partial intervention:  0.05 (95% CI -0.17, 026; p = 0.66), ES = 0.06 (3 months)  -0.13 (95% CI -0.38, 011; p = 0.28), ES =  -0.14 (1 year) | No statistically significant differences between groups for BMI z-score, with very small effect sizes between the full intervention or partial intervention and control group in all follow-ups. BMI z-score improved significantly (p = 0.019) between baseline and follow-up within the partial intervention. |
| **Healthy Together Victoria (HTV)**  **Strugnell et al. 2024 (28)**  **Strugnell et al. 2016 (29)** | Baseline and 2-years follow-up | Randomly selected schools:  Baseline: 11 communities (13 schools n = 1,741 analyzed for BMI)  Follow-up: 11 communities (23 schools n = 1,940 analyzed for BMI)  N varies for each outcome | Randomly selected schools:  Baseline: 12 communities (10 schools, n = 1,139 analyzed for BMI)  Follow-up: 12 communities (17 schools, n = 1,394 analyzed for BMI)  Schools were matched to intervention schools by sociodemographic index and prevalence of unhealthy weight among adults | 1) BMI z-score (WHO reference)  Baseline:  0.55 (95% CI 0.43, 0.66)  Follow-up:  0.56 (95% CI 0.46, 0.67)  Difference:  0.01 (95% CI -0.1, 0.13, p = 0.811)  2) Overweight/obesity prevalence (%)  Baseline:  34.4 (95% CI 30.4, 38.4)  Follow-up:  33.8 (95% CI 29.7, 38)  Difference:  -0.6 (95% CI -4.2, 3; p = 0.743) | 1) Baseline:  0.46 (95% CI 0.33, 0.59)  Follow-up:  0.54 (95% CI 0.42, 0.67)  Difference:  0.08 (95% CI -0.03, 0.2, p = 0.143)  2) Baseline:  29.6 (95% CI 25.9, 33.2)  Follow-up:  32.5 (95% CI 28.1, 36.9)  Difference:  2.9 (95% CI -0.7, 6.5; p = 0.116) | 1) -0.07 (95% CI -0.23, 0.09, p = 0.382)  2) -3.5 (95% CI -8.8, 1.8, p = 0.196) | No statistically significant intervention effect in BMI and overweight/obesity prevalence, but the effects were favorable for the intervention group. |
| **FLASH intervention**  **Van Dongen et al. 2022 (30)** | Baseline and each year until the end of the study (2 to 3 measurements per cohort) | 4 schools (n = 460 for BMI) | 4 schools (n = 460 for BMI) | 1) BMI z-score  Only effect sizes are reported | 1) Only effect sizes are reported | 1) Baseline to 1^st^ follow-up: -0.08 (95% CI -0.19, 0.03; p = 0.142)  Baseline to 2^nd^ follow-up:  -0.09 (95% CI -0.21, 0.03; p = 0.155)  Overall effect:  -0.09 (95% CI -0.19, 0.02; p = 0.096)  Adjusted for sex, educational level and migration background | No statistically significant intervention effects for BMI z-score (small differences stable over time), but the effects were favorable for the intervention. When sample was stratified by cohorts (A, B and C based on the year of recruitment), intervention effects in BMI z-score were statistically significant for cohort B (2017). |
| **CHIRPY DRAGON**  **Li et al. 2019 (31)**  **Li et al. 2017 (32)** | Baseline and 1-year follow-up | Baseline:  20 schools (n = 826 measured)  Follow-up: 20 schools (n = 794 analyzed) | Baseline:  20 schools (n = 804 measured)  Follow-up: 20 schools (n = 768 analyzed) | 1) BMI z-score (WHO 2007 Growth Charts)  Baseline:  -0.13 SD 1.30  Follow-up:  -0.35 SD 1.22  Difference:  -0.22  2) Overweight/obesity prevalence (%):  Baseline: NR  Follow-up: 15.5%  Difference: NA | 1) Baseline:  -0.13 SD 1.30  Follow-up:  -0.23 SD 1.34  Difference:  -0.10  2) Baseline: NR  Follow-up: 18.8%  Difference: NA | 1) Mean difference:  -0.13 (95% CI -0.26, -0.00; p = 0.048) (baseline adjusted analysis); -0.13 (95% CI -0.26, -0.01; p = 0.041) (further adjusted analysis)  2) OR: 0.53 (95% CI 0.27, 1.05; p = 0.067) (baseline adjusted analysis); 0.65 (95% CI 0.31, 1.36; p = 0.258) (further adjusted analysis)  Adjusted for baseline outcome and school clustering (baseline); baseline outcome, prespecified school-level, child-level sociodemographic and behavioral and measured sedentary time covariates (further analysis). | Statistically significant favorable intervention effect on reducing BMI z-score, but not significant for overweight/obesity prevalence. In the subgroup analysis, a larger effect was observed in girls and in children with overweight or obesity at baseline. |
| **Healthy Caregivers – Healthy Children (HC2)**  **Natale et al. 2021 (33)** | Baseline and 3 follow-up timepoints (at 1, 1.5 and 2 years) | Phase 1: 16 centers (n = 457)  Phase 2: 12 centers (n = 360) | Phase 1: 12 centers (n = 767)  Phase 2: 12 centers (n = 465) | 1) PBMI  Baseline: NR  Follow-up: NR  Difference:  All weight groups: -0.01 points  Healthy weight: 9 points; p = 0.0032 (Phase 1); 5 points; p = 0.050 (Phase 2)  Unhealthy weight: Increased; p value NR (Phase 2) | 1) Baseline: NR  Follow-up: NR  Difference:  All weight groups: 0.16 points  Healthy weight: 6 points; p = 0.027 (Phase 2)  Unhealthy weight: Increased; p = 0.023 (Phase 2) | 1) Phase 1:  0.01, p = 0.002 (all weight groups); p = 0.7212 (healthy weight); p = 0.0007 (unhealthy weight)  Phase 2: 0.16, p = 0.002 (all weight groups); p = 0.0042 (healthy weight); p = 0.2348 (unhealthy weight)  Adjusted for gender, race/ethnicity, age and clustering of students within centers. | No statistically significant changes over time. Statistically significant differences between control and intervention when weight groups are stratified. Mean child PBMI stayed healthy over the 2 years in both the control and intervention groups for phases 1 and 2 of the study. The university-based research team seemed to be more effective (phase 1) than the train-the-trainers approach (phase 2). |
| **WHO-STOPS Allender et al., 2021 (34)** | Baseline, 2-year and 4-year follow-up | Baseline:  5 communities (25 schools, n = 972 students)  Follow-up:  5 communities (25 schools, n = 1,041 at 2 years; 23 schools, n = 878 at 4 years) | Baseline:  5 communities (15 schools, n = 820)  Follow-up:  5 communities (23 schools, n = 1,370 students at 2 years; 21 schools, n = 1,259 at 4 years) | 1) BMI z-score  Baseline:  0.64 (95% CI 0.52-0.76)  Follow-up:  0.55 (95% CI 0.44-0.67) (year 2); 0.74 (95% CI 0.62-0.86) (year 4)  Difference:  -0.9 (year 2); 0.1 (year 4)  2) Overweight/obesity (%)  Baseline:  35.5 (95% CI 31.6 to 39.4)  Follow-up:  31.5 (95% CI 27.4 to 35.6) (year 2); 40.4 (95% CI 35.8 to 45.0) (year 4)  Difference:  -4 (year 2); 4.9 (year 4) | 1) Baseline:  0.60 (95% CI 0.46-0.73)  Follow-up:  0.60 (95% CI 0.48-0.73) (year 2); 0.60 (95% CI 0.48-0.72) (year 4)  Difference:  0.0 (year 2); 0.0 (year 4)  2) Baseline:  34.3 (95% CI 29.5 to 39.2)  Follow-up:  32.9 (95% CI 29.0 to 36.7) (year 2); 34.7 (95% CI 30.8-38.7) (year 4)  Difference:  -1.4 (year 2); 0.4 (year 4) | 1) 2-year follow-up:  −0.09 (95% CI −0.24, 0.06; p = 0.217)  4-year follow-up:  0.10 (95% CI −0.06, 0.25; p = 0.219)  2) 2-year follow-up:  −2.5 (95% CI −7.2, 2.2; p = 0.297)  4-year follow-up:  4.5 (95% CI −0.5, 9.4; p = 0.079)  Adjusted for school (clustering), group, wave (start year), interaction group x wave, school´s SES index, type of school | No statistically significant intervention effect for BMI and obesity/overweight prevalence in the four years. Statistically significant interaction effect between trial arm and wave (time). Positive effects  of the intervention were observed for some diet behaviors and some physical, psychosocial, and global  HRQoL. |
| **The Danish SoL Project**  **Buch-Andersen et al., 2021 (35)** | Baseline and 19-months follow-up | Baseline:  3 communities (4 childcare centers and 3 schools, n = 214 children)  Follow-up:  3 communities (4 childcare centers and 3 schools, n = 175 children analyzed) | Baseline:  3 communities (3 childcare centers and 3 schools, n = 238 children)  Follow-up:  3 communities (3 childcare centers and 3 schools, n = 170 children analyzed) | 1) BMI z-score (Danish 2014 growth reference)  Baseline:  Median 0.15 (IQR -0.58, 0.84)  Follow-up: Median 0.04 (IQR -0.61, 0.91)  Adjusted difference for age, parental education, household income and family status: -0.09 (95% CI 0.01, 0.17)  2) Overweight prevalence (%, IOTF cutoffs)  Baseline: 13%  Follow-up: 11%  Difference: -2%  3) Obesity prevalence (%)  Baseline: 2%  Follow-up: 3%  Difference: 1% | 1) Baseline:  Median 0.32 (IQR -0.32, 0.77)  Follow-up: Median 0.15 (IQR -0.35, 0.85)  Adjusted difference for age, parental education, household income and family status: -0.10 (95% CI -0.18, -0.02)  2) Baseline: 12%  Follow-up: 11%  Difference: -1%  3) Baseline: 0%  Follow-up: 0%  Difference: 0% | 1) 0.19 (95% CI 0.08, 0.30; p = 0.0013)  2) NR (no values were reported)  3) NR (no values were reported)  Adjusted for age, sex, parental education, household income and family status | Statistically significant differences between intervention and control groups in favor of the control group (BMI z-scores decreased in the control group and increased in the intervention group). No favorable effects of Project SoL on BMI z-scores and WC in children. |
| **NET-Works**  **French et al., 2023 (36)** | Baseline, 12, 24, 36 and 66 months follow-ups | Baseline:  12 primary care clinics (n = 265 children)  Follow-up:  12 primary care clinics (n = 242 at 12 months, 224 at 24 months, 235 at 36 months, 151 at 66 months) | Baseline:  12 primary care clinics (n = 269 children)  Follow-up:  12 primary clinics (n = 261 at 12 months, n = 257 at 24 months, n = 258 at 36 months, n = 187 at 66 months) | 1) BMI (kg/m^2^)  Baseline:  17.8 SD 2.2  Follow-up:  18.4 SD 3.3 (36 months); 20.8 SD 4.3 (66 months)  Difference:  0.6 (36 months); 3 (66 months)  2) BMI percentile  Baseline:  81.0 SD 15.2  Follow-up:  81.1 SD 18.8 (36 months); 80.6 SD 20.3 (66 months)  Difference:  0.1 (36 months); -0.4 (66 months)  3) BMI z-score  Baseline:  1.2 SD 1.0  Follow-up:  1.2 SD 0.9 (36 months); 1.1 SD 0.9 (66 months)  Difference: 0 (36 months); -0.1 (66 months)  4) Obesity prevalence (%)  Baseline:  24.9  Follow-up:  28.9 (36 months); 33 (66 months)  Difference:  4 (36 months); 8.1 (66 months) | 1) Baseline:  17.4 SD 1.3  Follow-up:  18.3 SD 2.7 (36 months); 21.2 SD 4.3 (66 months)  Difference:  0.9 (36 months); 3.8 (66 months)  2) Baseline:  82.5 SD 13.3  Follow-up:  81.9 SD 18.1 (36 months); 80.8 SD 22.5 (66 months)  Difference:  0.6 (36 months); -1.7 (66 months)  3) Baseline:  1.1 SD 0.7  Follow-up:  1.2 SD 0.8 (36 months); 1.2 SD 0.9 (66 months)  Difference:  0.1 (36 months); 0.1 (66 months)  4) Baseline:  20.5  Follow-up:  31.8 (36 months); 40 (66 months)  Difference:  11.3 (36 months); 19.5 (66 months) | 1) −0.19 (95% CI −0.64, 0.26) (36 months); −0.38 (95% CI −1.13, 0.37) (66 months)  2) −0.33 (95% CI −3.93, 3.26) (36 months); 1.15 (95% CI −3.16, 5.45) (66 months)  3) −0.07 (95% CI −0.23, 0.08) (36 months); −0.02 (95% CI −0.19, 0.15) (66 months)  4) OR: 0.68 (95% CI 0.41, 1.14) (36 months); 0.71 (95% CI 0.43, 1.15) (66 months)  All differences had a p value > 0.05. All mean differences were adjusted for child, gender and baseline value. | No statistically significant intervention effects at 66 months for BMI measures and obesity prevalence.  At 66 months, the intervention had small but statistically significant effects on reducing LDL cholesterol, energy intake, and increasing vigorous physical activity. No significant differences were observed for other cardiometabolic outcomes, physical activity levels, or sedentary behaviors. |
| **Communities for Healthy Living**  **Gago et al., 2023 (37)** | Baseline (fall), follow-up (spring) in each intervention (academic) year | 16 Head Start programs (n=3,368 children enrolled, n = 2,579 children analyzed, n = 454 parents analyzed)  Same programs in intervention and control, but different starting points | 10 Head Start programs (n = 1,631 children enrolled, n = 1,318 children analyzed, n = 501 parents analyzed)  Same programs in intervention and control, but different starting points | N = 1,274  1) BMI z-score, only children (CDC growth charts)  Baseline:  0.58 SD 1.26  Follow-up:  0.62 (self-calculation)  Difference:  0.04 SD 0.44  2) Modified BMI z-score (adjusted version of BMI z-score), only children  Baseline:  0.57 SD 1.42  Follow-up:  0.62 (self-calculation)  Difference:  0.05 SD 0.46 | N = 2,476  1) Baseline:  0.64 SD 1.20  Follow-up:  0.63 (self-calculation)  Difference:  −0.01 SD 0.42  2) Baseline:  0.63 SD 1.40  Follow-up:  0.61 (self-calculation)  Difference:  −0.02 SD 0.45 | 1) 0.06 (95% CI 0.02, 0.09; p < 0.01) unadjusted;  0.06 (95% CI 0.02, 0.10; p < 0.01) adjusted  2) 0.07 (95% CI 0.03, 0.10; p <0.001) unadjusted;  0.07 (95% CI 0.03, 0.12; p < 0.01) adjusted  Adjusted for parent race and ethnicity, educational level and household employment status | Small statistically significant mean increase per year in BMI z-scores in the intervention compared to the control groups. 34.4% to 33.8%  The intervention improved child health behaviors, increasing the odds of meeting recommendations for sugar-sweetened beverages, water intake, and screen time. Parents in the intervention group showed a significant increase in empowerment, while other parental outcomes did not differ significantly. |

Legend: *Differences were reported directly from the studies or calculated from given data if not reported in the studies. Abbreviations: *aOR – adjusted Odds Ratio, BMI – body mass index, BMI z-score – BMI standardized score, CDC – Center for Disease Control, CI – confidence interval, Comm. – community, Fam – family, HRQoL – health-related quality of life, IOTF – International Obesity Task Force, IQR – interquartile range, NA – not applicable, NR – not reported, PBMI – body mass index percentile, RR – relative risk SD – standard deviation, SE – standard error, SES – socioeconomic status, WC – waist circumference, WHO – World Health Organization, ys – years*

Table S5. Risk of bias assessment of included studies.

| Questions/  Projects | Shape Up Somerville [37,45,48] | OPAL [33] | POIBC-TCHP [29] | BAEW [34,49,50] | HCP-BAEW [31] | IDEFICS [38,47] | VIASANO [39] | Aventuras para Niños [30] | EWBA [36] | Ma’alahi Youth [40] | Healthy Youth Healthy Communities [41] | Healthy Alberta Communities [44,51] | Zhou et al. [42] | Romp & Chomp [35] | APPLE [43] | POP [32] |
| --- | --- | --- | --- | --- | --- | --- | --- | --- | --- | --- | --- | --- | --- | --- | --- | --- |
| Was there a comparison? |  |  |  |  |  |  |  |  |  |  |  |  |  |  |  |  |
| Between two or more groups of clusters receiving different interventions? | Y | Y | Y | Y | Y | Y | Y | Y | Y | Y | Y | Y ^6^ | Y | Y | Y | Y |
| Within the same group of clusters over time? | Y | Y | Y | Y | Y | Y | Y | Y | Y | Y | Y | Y | Y | Y | Y | Y |
|  |  |  |  |  |  |  |  |  |  |  |  |  |  |  |  |  |
| Were clusters allocated to groups by: |  |  |  |  |  |  |  |  |  |  |  |  |  |  |  |  |
| Concealed randomization? | N | N | N | N | N | N | N | NC | N | N | N | N | N | N | N | N |
| Quasi-randomization? | N | Y | N | Y | N | N | N | Y ^3^ | N | N | N | N | N | N | N | N |
| By other action of researchers? | Y | N | N | Y | Y | Y | N | N | Y ^4^ | Y ^4^ | N | Y ^4^ | Y | Y ^8^ | Y^9^ | Y^10^ |
| Time differences? | N | N | N | N | N | N | N | N | N | N | N | N | N | N | N | N |
| Location differerences? | Y | Y | Y | Y | Y | N | N | N | N | N | N | Y | Y ^7^ | N | N | N |
| Policy/public health decisions? | N | N | N | N | N | Y | N | N | N | N | N | N | N | N | N | N |
| Cluster preferences? | N | N | N | N | N | N | N | N | N | N | N | N | N | N | N | N |
| Some other processes? (specify) | N | N | N | N | N | Y ^1^ | Y ^2^ | N | N | N | Y ^5^ | N | N | N | N | N |
|  |  |  |  |  |  |  |  |  |  |  |  |  |  |  |  |  |
| Which parts of the study were prospective: |  |  |  |  |  |  |  |  |  |  |  |  |  |  |  |  |
| Identification of participating clusters? | Y | Y | Y | Y | Y | Y | Y | Y | Y | Y | Y | Y | Y | Y | Y | Y |
| Assessment of baseline and allocation to intervention? | Y | Y | Y | Y | Y | Y | Y | Y | Y | Y | Y | Y | Y | Y | Y | Y |
| Assessment of outcomes? | Y | Y | Y | Y | Y | Y | Y | Y | Y | Y | Y | Y | Y | Y | Y | Y |
| Generation of hypotheses? | NC | Y | NR | NR | NR | Y | NR | Y | NR | NR | NR | NR | NR | Y | NR | NR |
|  |  |  |  |  |  |  |  |  |  |  |  |  |  |  |  |  |
| On what variables was comparability between groups assessed? |  |  |  |  |  |  |  |  |  |  |  |  |  |  |  |  |
| Potential confounders? | Y | N | Y | Y | Y | Y | Y | Y | NC | Y | Y | NC | Y | NC | Y | Y |
| Baseline assessment of outcome variables? | Y | Y | Y | Y | Y | Y | Y | Y | Y | Y | Y | Y | Y | Y | Y | Y |

| Questions/  Projects | SuperFIT Project (26, 27) | The Healthy Together Victoria (28, 29) | FLASH intervention (30) | CHIRPY DRAGON (31, 32) | Healthy Caregivers-Healthy Children (33) | Allender et al., 2021 (34) | Buch-Andersen et al., 2021 (35) | French et al., 2023 (36) | Gago et al., 2023 (37) |
| --- | --- | --- | --- | --- | --- | --- | --- | --- | --- |
| *Was there a comparison?* |  |  |  |  |  |  |  |  |  |
| Between two or more groups of clusters receiving different interventions? | Y | Y | Y | Y | Y | Y | Y | NA | Y |
| Within the same group of clusters over time? | Y | Y | Y | Y | Y | Y | Y | NA | Y |
|  |  |  |  |  |  |  |  |  |  |
| *Were clusters allocated to groups by:* |  |  |  |  |  |  |  |  |  |
| Concealed randomization? | N | N | N | Y | N | NC | N | NA | Y |
| Quasi-randomization? | N | Y^2^ | N | N | Y | N | Y | NA | N |
| By other action of researchers? | N | N | N | N | N | Y^11^ | N | NA | N |
| Time differences? | N | N | N | N | N | N | N | NA | N |
| Location differerences? | N | N | N | N | N | N | N | NA | N |
| Policy/public health decisions? | N | N | N | N | N | N | N | NA | N |
| Cluster preferences? | N | N | N | N | N | N | N | NA | N |
| Some other processes? (specify) | Y^1^ | N | Y^3^ | N | N | N | N | NA | N |
|  |  |  |  |  |  |  |  |  |  |
| *Which parts of the study were prospective:* |  |  |  |  |  |  |  |  |  |
| Identification of participating clusters? | Y | Y | Y |  | Y | Y | Y | Y | Y |
| Assessment of baseline and allocation to intervention? | Y | Y | Y |  | Y | Y | Y | Y | Y |
| Assessment of outcomes? | Y | Y | Y |  | Y | Y | Y | Y | Y |
| Generation of hypotheses? | Y | Y | Y |  | Y | Y | Y | Y | Y |
|  |  |  |  |  |  |  |  |  |  |
| *On what variables was comparability between groups assessed?* |  |  |  |  |  |  |  |  |  |
| Potential confounders? | Y | Y | Y^4^ | Y | Y | Y^12^ | Y | Y | Y |
| Baseline assessment of outcome variables? | Y | Y | Y | Y | Y | Y^12^ | Y | Y | Y |

Abbreviations: *N – No, NC – not clear, NR – not reported, Y – Yes*

^1^ Similar socioeconomic status

^2^ Matched randomization of clusters by sociodemographic index and prevalence of unhealthy weight among adults

^3^ Intervention schools recruited and were eligible to participate if they would commit to the intervention and evaluation of 4 years. Controls were matched based on surroundings, size, educational streams, familiarity with the Healthy School Approach

^4^ Potential residual confounding, adjustment only for sex, educational level and immigration background

^5^ Intervention community selected because they had conducted an ANGELO workshop, control matched by ethnic profile

^6^ The samples were not the same for all measures, control was national data

^7^ Intervention community was selected because of the close distance to the research institution

^8^ Sample selected because of electronic anthropometric data available

^9^ Intervention and control communities selected because of their geographical separation and similar sociodemographic variables

^10^ Communities were matched and distributed to control and intervention groups based on socioeconomic status

^11^ Initial stepped-wedge design was not completed due to natural disaster disrupting the trial

^12^ No correction for multiple comparisons was used.

Source: Cochrane assessment tool of risk of bias for cluster non-randomized studies (https://handbook-5-1.cochrane.org/chapter_13/box_13_4_a_user_guide_for_data_collection_study_assessment.htm)

References

1. Coffield E, Nihiser AJ, Sherry B, Economos CD. Shape Up Somerville: change in parent body mass indexes during a child-targeted, community-based environmental change intervention. Am J Public Health. 2015;105(2):e83-9.

2. Economos CD, Hyatt RR, Goldberg JP, Must A, Naumova EN, Collins JJ, et al. A community intervention reduces BMI z-score in children: Shape Up Somerville first year results. Obesity (Silver Spring). 2007;15(5):1325-36.

3. Economos CD, Hyatt RR, Must A, Goldberg JP, Kuder J, Naumova EN, et al. Shape Up Somerville two-year results: a community-based environmental change intervention sustains weight reduction in children. Prev Med. 2013;57(4):322-7.

4. Bell L, Ullah S, Leslie E, Magarey A, Olds T, Ratcliffe J, et al. Changes in weight status, quality of life and behaviours of South Australian primary school children: results from the Obesity Prevention and Lifestyle (OPAL) community intervention program. BMC Public Health. 2019;19(1):1338.

5. Gomez SF, Casas Esteve R, Subirana I, Serra-Majem L, Fletas Torrent M, Homs C, et al. Effect of a community-based childhood obesity intervention program on changes in anthropometric variables, incidence of obesity, and lifestyle choices in Spanish children aged 8 to 10 years. Eur J Pediatr. 2018;177(10):1531-9.

6. Ko LK, Rillamas-Sun E, Bishop S, Cisneros O, Holte S, Thompson B. Together We STRIDE: A quasi-experimental trial testing the effectiveness of a multi-level obesity intervention for Hispanic children in rural communities. Contemp Clin Trials. 2018;67:81-6.

7. Swinburn B, Malakellis M, Moodie M, Waters E, Gibbs L, Millar L, et al. Large reductions in child overweight and obesity in intervention and comparison communities 3 years after a community project. Pediatr Obes. 2014;9(6):455-62.

8. Johnson BA, Kremer PJ, Swinburn BA, de Silva-Sanigorski AM. Multilevel analysis of the Be Active Eat Well intervention: environmental and behavioural influences on reductions in child obesity risk. Int J Obes (Lond). 2012;36(7):901-7.

9. Sanigorski AM, Bell AC, Kremer PJ, Cuttler R, Swinburn BA. Reducing unhealthy weight gain in children through community capacity-building: results of a quasi-experimental intervention program, Be Active Eat Well. Int J Obes (Lond). 2008;32(7):1060-7.

10. Bolton KA, Kremer P, Gibbs L, Waters E, Swinburn B, de Silva A. The outcomes of health-promoting communities: being active eating well initiative-a community-based obesity prevention intervention in Victoria, Australia. Int J Obes (Lond). 2017;41(7):1080-90.

11. Gittelsohn J, Jock B, Redmond L, Fleischhacker S, Eckmann T, Bleich SN, et al. OPREVENT2: Design of a multi-institutional intervention for obesity control and prevention for American Indian adults. BMC Public Health. 2017;17(1):105.

12. Verbestel V, De Henauw S, Barba G, Eiben G, Gallois K, Hadjigeorgiou C, et al. Effectiveness of the IDEFICS intervention on objectively measured physical activity and sedentary time in European children. Obes Rev. 2015;16 Suppl 2:57-67.

13. De Henauw S, Huybrechts I, De Bourdeaudhuij I, Bammann K, Barba G, Lissner L, et al. Effects of a community-oriented obesity prevention programme on indicators of body fatness in preschool and primary school children. Main results from the IDEFICS study. Obes Rev. 2015;16 Suppl 2:16-29.

14. Vinck J, Brohet C, Roillet M, Dramaix M, Borys JM, Beysens J, et al. Downward trends in the prevalence of childhood overweight in two pilot towns taking part in the VIASANO community-based programme in Belgium: data from a national school health monitoring system. Pediatr Obes. 2016;11(1):61-7.

15. Ayala GX, Ibarra L, Binggeli-Vallarta A, Moody J, McKenzie TL, Angulo J, et al. Our Choice/Nuestra Opcion: the Imperial County, California, Childhood Obesity Research Demonstration study (CA-CORD). Child Obes. 2015;11(1):37-47.

16. Crespo NC, Elder JP, Ayala GX, Slymen DJ, Campbell NR, Sallis JF, et al. Results of a multi-level intervention to prevent and control childhood obesity among Latino children: the Aventuras Para Ninos Study. Ann Behav Med. 2012;43(1):84-100.

17. Pettman T, Magarey A, Mastersson N, Wilson A, Dollman J. Improving weight status in childhood: results from the eat well be active community programs. Int J Public Health. 2014;59(1):43-50.

18. Fotu KF, Millar L, Mavoa H, Kremer P, Moodie M, Snowdon W, et al. Outcome results for the Ma'alahi Youth Project, a Tongan community-based obesity prevention programme for adolescents. Obes Rev. 2011;12 Suppl 2:41-50.

19. Kremer P, Waqa G, Vanualailai N, Schultz JT, Roberts G, Moodie M, et al. Reducing unhealthy weight gain in Fijian adolescents: results of the Healthy Youth Healthy Communities study. Obes Rev. 2011;12 Suppl 2:29-40.

20. Lytvyak E, Olstad DL, Schopflocher DP, Plotnikoff RC, Storey KE, Nykiforuk CI, et al. Impact of a 3-year multi-centre community-based intervention on risk factors for chronic disease and obesity among free-living adults: the Healthy Alberta Communities study. BMC Public Health. 2016;16:344.

21. Raine KD, Plotnikoff R, Schopflocher D, Lytvyak E, Nykiforuk CI, Storey K, et al. Healthy Alberta Communities: impact of a three-year community-based obesity and chronic disease prevention intervention. Prev Med. 2013;57(6):955-62.

22. Zhou Z, Ren H, Yin Z, Wang L, Wang K. A policy-driven multifaceted approach for early childhood physical fitness promotion: impacts on body composition and physical fitness in young Chinese children. BMC Pediatr. 2014;14:118.

23. de Silva-Sanigorski AM, Bell AC, Kremer P, Nichols M, Crellin M, Smith M, et al. Reducing obesity in early childhood: results from Romp & Chomp, an Australian community-wide intervention program. Am J Clin Nutr. 2010;91(4):831-40.

24. Taylor RW, McAuley KA, Barbezat W, Strong A, Williams SM, Mann JI. APPLE Project: 2-y findings of a community-based obesity prevention program in primary school age children. Am J Clin Nutr. 2007;86(3):735-42.

25. De Coen V, De Bourdeaudhuij I, Vereecken C, Verbestel V, Haerens L, Huybrechts I, et al. Effects of a 2-year healthy eating and physical activity intervention for 3-6-year-olds in communities of high and low socio-economic status: the POP (Prevention of Overweight among Pre-school and school children) project. Public Health Nutr. 2012;15(9):1737-45.

26. van de Kolk I, Gerards S, Harms LSE, Kremers SPJ, van Dinther-Erkens A, Snellings M, et al. Study Protocol for the Evaluation of "SuperFIT", a Multicomponent Nutrition and Physical Activity Intervention Approach for Preschools and Families. Int J Environ Res Public Health. 2020;17(2).

27. van de Kolk I, Gerards S, Harms LSE, Kremers SPJ, Gubbels JS. The Effects of a Comprehensive, Integrated Obesity Prevention Intervention Approach (SuperFIT) on Children's Physical Activity, Sedentary Behavior, and BMI Z-Score. Int J Environ Res Public Health. 2019;16(24).

28. Strugnell C, Orellana L, Crooks N, Malakellis M, Morrissey B, Rennie C, et al. Healthy together Victoria and childhood obesity study: effects of a large scale, community-based cluster randomised trial of a systems thinking approach for the prevention of childhood obesity among secondary school students 2014-2016. BMC Public Health. 2024;24(1):355.

29. Strugnell C, Millar L, Churchill A, Jacka F, Bell C, Malakellis M, et al. Healthy together Victoria and childhood obesity-a methodology for measuring changes in childhood obesity in response to a community-based, whole of system cluster randomized control trial. Arch Public Health. 2016;74:16.

30. van Dongen BM, de Vries IM, Ridder MAM, de Boer M, Steenhuis IHM, Renders CM. Building community capacity to stimulate physical activity and dietary behavior in Dutch secondary schools: Evaluation of the FLASH intervention using the REAIM framework. Front Public Health. 2022;10:926465.

31. Li B, Pallan M, Liu WJ, Hemming K, Frew E, Lin R, et al. The CHIRPY DRAGON intervention in preventing obesity in Chinese primary-school--aged children: A cluster-randomised controlled trial. PLoS Med. 2019;16(11):e1002971.

32. Li B, Liu WJ, Adab P, Pallan M, Hemming K, Frew E, et al. Cluster-randomised controlled trial to assess the effectiveness and cost-effectiveness of an obesity prevention programme for Chinese primary school-aged children: the CHIRPY DRAGON study protocol. BMJ Open. 2017;7(11):e018415.

33. Natale RA, Atem F, Weerakoon S, Lebron C, Mathew MS, Sardinas K, et al. An Implementation Approach Comparison of a Child Care Center-Based Obesity Prevention Program. J Dev Behav Pediatr. 2021;42(2):135-45.

34. Allender S, Orellana L, Crooks N, Bolton KA, Fraser P, Brown AD, et al. Four-Year Behavioral, Health-Related Quality of Life, and BMI Outcomes from a Cluster Randomized Whole of Systems Trial of Prevention Strategies for Childhood Obesity. Obesity (Silver Spring). 2021;29(6):1022-35.

35. Buch-Andersen T, Eriksson F, Bloch P, Glümer C, Mikkelsen BE, Toft U. The Danish SoL Project: Effects of a Multi-Component Community-Based Health Promotion Intervention on Prevention of Overweight among 3-8-Year-Old Children. Int J Environ Res Public Health. 2021;18(16).

36. French SA, Kunin-Batson AS, Sherwood NE, Berge JM, Shanley R. NET-Works paediatric obesity prevention trial: 66 month outcomes. Pediatr Obes. 2023;18(8):e13055.

37. Gago C, Aftosmes-Tobio A, Beckerman-Hsu JP, Oddleifson C, Garcia EA, Lansburg K, et al. Evaluation of a cluster-randomized controlled trial: Communities for Healthy Living, family-centered obesity prevention program for Head Start parents and children. Int J Behav Nutr Phys Act. 2023;20(1):4.
